# Clinical entity augmented retrieval for clinical information extraction

**DOI:** 10.1038/s41746-024-01377-1

**Published:** 2025-01-19

**Authors:** Ivan Lopez, Akshay Swaminathan, Karthik Vedula, Sanjana Narayanan, Fateme Nateghi Haredasht, Stephen P. Ma, April S. Liang, Steven Tate, Manoj Maddali, Robert Joseph Gallo, Nigam H. Shah, Jonathan H. Chen

**Affiliations:** 1 Stanford University School of Medicine, Stanford, CA USA; 2 Department of Biomedical Data Science, Stanford, CA USA; 3 Poolesville High School, Poolesville, MD USA; 4 Stanford Center for Biomedical Informatics Research, Stanford, CA USA; 5 Division of Hospital Medicine, Stanford University School of Medicine, Stanford, CA USA; 6 Division of Clinical Informatics, Stanford University School of Medicine, Stanford, CA USA; 7 Department of Psychiatry and Behavioral Sciences, Stanford University School of Medicine, Stanford, CA USA; 8 Division of Pulmonary, Allergy, and Critical Care Medicine, Stanford University School of Medicine, Stanford, CA USA; 9 Center for Innovation to Implementation, VA Palo Alto Healthcare System, Menlo Park, CA USA; 10 Department of Health Policy, Stanford University, Stanford, CA USA; 11 Technology and Digital Solutions, Stanford Healthcare, Palo Alto, USA; 12 Clinical Excellence Research Center, Stanford University School of Medicine, Stanford, CA USA; 13 Department of Medicine, Stanford, CA USA

**Keywords:** Machine learning, Data mining

## Abstract

Large language models (LLMs) with retrieval-augmented generation (RAG) have improved information extraction over previous methods, yet their reliance on embeddings often leads to inefficient retrieval. We introduce CLinical Entity Augmented Retrieval (CLEAR), a RAG pipeline that retrieves information using entities. We compared CLEAR to embedding RAG and full-note approaches for extracting 18 variables using six LLMs across 20,000 clinical notes. Average F1 scores were 0.90, 0.86, and 0.79; inference times were 4.95, 17.41, and 20.08 s per note; average model queries were 1.68, 4.94, and 4.18 per note; and average input tokens were 1.1k, 3.8k, and 6.1k per note for CLEAR, embedding RAG, and full-note approaches, respectively. In conclusion, CLEAR utilizes clinical entities for information retrieval and achieves >70% reduction in token usage and inference time with improved performance compared to modern methods.

## Introduction

Free-text notes in electronic health records (EHRs) are rich with data not found within structured fields, like symptoms, diagnoses, disease course, social determinants of health, family history, and patient perspectives^1,2^. The ability to process this data unlocks various important research and quality improvement use cases, including cohort selection^3^, phenotyping^4^, observational data analysis^5^, and predictive modeling^6^.

Despite the amount of valuable information in EHRs, extracting information from clinical notes remains challenging^7,8^. Clinical information extraction comprises several tasks, including named entity recognition (NER) (e.g., recognizing “t2dm” as type II diabetes mellitus)^9^, sense disambiguation (e.g., understanding “mi” as “myocardial infarction” or “mitral insufficiency” depending on the context)^10^, and relation extraction (e.g., linking a symptom with medication if reported as a side-effect)^11^.

The simplest clinical information extraction approaches use rules and dictionaries to identify entities of interest^12,13^, such as diagnosis codes like the International Classification of Diseases (ICD). In a 2018 review of 263 clinical information extraction methods, 65% were rule-based^8^. These systems are interpretable, easy to deploy, and achieve reasonable performance on many tasks^12^. However, structured fields like diagnosis codes are unable to fully capture a patient’s medical history in the current state. For example, despite a recent increase in the use of diagnosis codes to represent social determinants of health, they remain underutilized and often miss crucial contextual details only found in the unstructured text of EHRs^14,15^. Moreover, for many conditions, such as cancer, ICD codes do not reflect the true source of diagnosis; in these cases, pathology reports are the gold standard^16,17^. Natural language processing methods are therefore necessary to extract these insights, allowing for a more comprehensive understanding of patient health. Additionally, hard-coded rules and word lists fail to capture the wide variation in clinical language, including synonyms and abbreviations, and miss nuanced descriptions in EHR notes^18^.

Supervised machine learning approaches that take in a labeled dataset can recognize more complex linguistic relationships than rules- or dictionary-based methods. Neural network architectures like bi-directional Long Short-Term Memory networks (LSTMs) are well suited for sequence data-based tasks like NER, given their ability to learn relationships between a token and its neighbor tokens in either direction^19^. For example, Stanza^20,21^, a widely used Python library for NER, uses a bi-directional LSTM with a Conditional Random Field trained on the 2010 i2b2/VA dataset^22^. A disadvantage of machine learning approaches is that they often require large, labeled training datasets, which can be time-consuming and expensive to obtain. Weak supervision offers an alternative to human-labeled data, where programmatic labeling functions are used to automatically assign “weak” labels. Although the quality of weak labels is lower than human labels, training on a large number of weak labels has been shown to outperform training on a small number of human labels. Labeling functions can be manually curated or sourced from ontologies or smaller models. For example, TROVE uses ontologies like the Unified Medical Language System (UMLS) to create labeling functions and uses these weak labels to fine-tune a BERT-based model for identifying symptoms and risk factors for COVID-19^23^.

Pre-trained deep learning models like BERT use multidimensional embeddings learned from large, unlabeled corpuses. These embeddings represent semantic information that can be used as features for a variety of downstream tasks. For instance, fine-tuned BERT-based models have been employed for tasks including named entity recognition, assertion status determination, sense disambiguation, and relation extraction^24,25^, and are often adapted to specific clinical domains, like radiology^26,27^. Although these models can be fine-tuned to perform tasks like diagnostic code assignment, treatment assignment^28^, and open-ended reasoning^29–32^, decoder-only models are typically better equipped for this task.

Recently, large language models (LLMs) trained with transformer-based architectures on large unlabeled text corpuses have demonstrated impressive performance on both information extraction and natural language understanding tasks, such as information extraction (e.g., “does this patient have diabetes?”)^33^, text summarization (e.g., “summarize this patient’s history”)^34^, and conversational capabilities (e.g., “draft a response to this patient’s message”)^35^. One advantage of LLMs is their “few-shot” and “zero-shot” prompting capabilities, enabling them to accomplish tasks with few to no labeled examples—tasks that previously required training or fine-tuning separate models with labeled datasets^36–38^. Recent work has used LLMs to extract clinical variables from EHR notes, including social determinants of health, medications, and postpartum hemorrhage^4,39,40^. While LLMs show great promise in clinical information retrieval, they face several limitations. For instance, the length of patient notes can surpass an LLM’s context window—the amount of text that can be passed into the model. Naive approaches like truncation or selecting only documents that fit within the context window risk excluding valuable information^4,39,41^. Dividing a note into smaller chunks with adjoining strides can address context window limits but still requires multiple LLM queries per patient, which can be computationally expensive. In addition, LLM performance has been shown to degrade on reasoning tasks as input length increases, even on models with large context windows^42–45^, suggesting that inputting long EHR excerpts containing extraneous information can reduce performance.

Retrieval-augmented generation (RAG) attempts to address this limitation by retrieving and appending query-relevant information to the input context. The retriever typically uses an encoder model to represent both the query and reference information in embedding space and retrieve information whose embeddings are close to that of the query^46^. Some RAG workflows embed small chunks of text that can fit within the model’s context window and store those embeddings in a database for downstream retrieval. Other approaches, mostly explored in the general domain, involve embedding and retrieving fact triplets from knowledge graphs^47–49^.

An important challenge in RAG-based methods is ensuring that the retrieved information is relevant to the query and does not contain extraneous information that can hinder LLM reasoning and add to inference costs^50,51^.

To address the above limitations of LLMs for clinical information extraction, we propose CLinical Entity Augmented Retrieval (CLEAR), a RAG pipeline that retrieves note chunks containing clinical entities relevant to the input query. We hypothesized that retrieval based on relevant clinical entities would lead to more efficient and relevant information retrieval compared to RAG approaches based on note chunk embeddings. We make three contributions. First, we validate the entity recognition and entity selection steps of the CLEAR pipeline, which identify clinical entities in clinical notes and select a subset relevant to the input query. Second, we compare CLEAR to a RAG approach that embeds note chunks and a full-note retrieval approach in performing information extraction for 18 clinical variables. Third, we explore the feasibility of using CLEAR to generate labels to fine-tune a BERT-sized model in performing information extraction. We conduct all experiments on two real-world EHR-derived datasets that include labels for substance use (e.g., alcohol dependence, tobacco dependence), mental health (e.g., attention-deficit/hyperactivity disorder [ADHD], bipolar disorder, depression), social determinants of health (e.g., homelessness, unemployment), and chest radiograph findings (e.g., pneumonia, cardiomegaly).

## Results

### Inter-rater reliability

In the Stanford MOUD dataset, the unweighted Cohen’s Kappa value was 0.86 (95% CI: 0.79-0.93). In the CheXpert dataset, the unweighted Cohen’s Kappa was 0.93 (95% CI: 0.88–0.98). These values indicate excellent agreement between annotators.

### NER and entity selection evaluation

Zero-shot NER using Flan-T5 identified 1269 out of 1382 entities (96% sensitivity) in the NCBI disease dataset and 440 out of 450 entities (99% sensitivity) in the Stanford MOUD dataset. We measured to what extent ontology and LLM augmentation recover entities missed in the NER step (false negatives) by using the UMLS ontology and GPT-4 to generate synonyms as if they were the target entity. This augmentation step increases sensitivity to 99% and 100% in the NCBI disease dataset and Stanford MOUD dataset, respectively, indicating that even if the target entity is missed during NER, it is very likely to be detected through ontology and LLM augmentation (Supplementary Table 1). The performance of each of the four zero-shot NER prompts on the Stanford MOUD dataset is detailed in Supplementary Table 2. We report the classification of false negatives for this analysis in Supplementary Table 3.

**Fig. 1:**
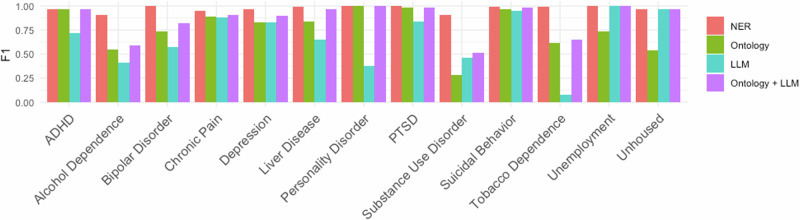
CLEAR information retrieval ablation F1 scores on Stanford MOUD dataset. F1 scores for information retrieval using NER, Ontology, LLM augmentation, or Ontology + LLM Augmentation on the Stanford MOUD Dataset. F1 scores were calculated for all 13 variables using GPT-4.

We studied the impact of the initial NER step on the overall performance of CLEAR. Overall, removing NER from CLEAR and relying only on ontology and LLM augmentation hurts downstream information extraction task performance, resulting in a 0.11 decrease in F1 across all 13 variables in the Stanford MOUD dataset (0.86 without NER vs. 0.97 with NER). For unhoused, personality disorder, ADHD, PTSD, suicidal behavior, liver disease, and unemployment, removing NER resulted in an F1 drop of ≤0.02. For other variables, removing NER led to a drop in F1 from 0.18 (bipolar disorder) to 0.40 (substance use disorder) (Fig. 1 and Supplementary Table 4). This suggests that for several variables, LLMs and ontologies do not capture the natural variation in clinical variables as effectively as NER. For a full list of high-yield terms missed by Ontology+LLM augmentations, refer to Supplementary Table 5.

### Information extraction evaluation

On the Stanford MOUD Dataset, the average F1 score across all 13 variables and all 6 models was 0.90, ranging from 0.78 (Med42) and 0.97 (GPT-4) across models. GPT-4 had the highest F1 score for 10 out of 13 variables. F1 scores across variables ranged from 0.61 (Med42 on depression) to 1.00 (GPT-4 on personality disorder, bipolar disorder, PTSD, and unemployment; Llama-3 on unhoused; Flan-T5 on unemployment). On the CheXpert Dataset, the average F1 score across all 5 test sets for all 6 models was 0.96, ranging from 0.91 (Flan-UL2) and 0.98 (Flan-T5 and Mixtral). Flan-T5 had the highest F1 score for 3 out of 5 variables. F1 scores across variables ranged from 0.90 (Med42 on pneumothorax) to 1.00 (Flan-T5 on cardiomegaly and pleural effusion) (Table 1). A full breakdown of CLEAR, chunk embedding, and full-note performance per variable is reported in Supplementary Table 6.

**Table 1:** CLEAR F1 scores for information extraction on the Stanford MOUD and CheXpert datasets

Variable	Flan-T5	Flan-UL2	GPT-4	Med42	Llama-3	Mixtral	Range	Fine-tuned BERT
CheXpert								
Cardiomegaly	1.00	0.96	0.95	0.97	0.95	0.99	1.00–0.95	0.95
Pulmonary edema	0.98	0.96	0.96	0.91	0.96	0.98	0.98–0.91	0.97
Pleural effusion	1.00	0.84	0.97	0.97	0.98	0.98	1.00–0.84	0.89
Pneumonia	0.95	0.84	0.99	0.88	0.94	0.95	0.99–0.84	0.94
Pneumothorax	0.98	0.95	0.99	0.90	0.97	0.98	0.99–0.90	0.96
Average	0.98	0.91	0.97	0.93	0.96	0.98	0.98–0.91	0.94
Stanford MOUD								
Depression	0.86	0.87	0.97	0.61	0.88	0.93	0.97–0.61	0.91
Alcohol dependence	0.85	0.81	0.91	0.69	0.74	0.75	0.91–0.69	0.91^a^
Substance use disorder	0.89	0.88	0.91	0.71	0.84	0.94	0.94–0.71	0.87
Unhoused	0.97	0.97	0.97	0.96	1.00	0.97	1.00–0.96	0.94
Tobacco dependence	0.95	0.98	0.99	0.70	0.90	0.92	0.99–0.70	0.90
Personality disorder	0.81	0.90	1.00	0.67	0.97	0.86	1.00–0.67	0.95
Bipolar disorder	0.90	0.94	1.00	0.91	0.89	0.94	1.00–0.89	0.90
PTSD	0.95	0.95	1.00	0.89	0.96	0.94	1.00–0.89	0.85
ADHD	0.94	0.97	0.97	0.77	0.87	0.84	0.97–0.77	0.77
Suicidal behavior	0.96	0.95	0.99	0.83	0.91	0.97	0.99–0.83	0.87
Liver disease	0.82	0.97	0.99	0.62	0.81	0.94	0.99–0.62	0.89
Chronic pain	0.95	0.97	0.95	0.88	0.94	0.94	0.97–0.88	0.98^a^
Unemployment	1.00	0.98	1.00	0.88	0.84	0.95	1.00–0.84	0.98
Average	0.91	0.93	0.97	0.78	0.89	0.91	0.97–0.78	0.90

^a^Fine-tuned BERT F1 score higher than the trainer model’s F1 score on the same held-out test set.

We used CLEAR to label a dataset to fine-tune a Bio+Clinical BERT for information extraction of the 13 variables in the Stanford MOUD dataset and 5 variables in the CheXpert dataset. Within the 13 Stanford MOUD Dataset classifiers, two showed perfect discrimination on the test set (AUC = 1). The “suicidal behavior” classifier had the lowest AUC (0.83). Within the CheXpert Dataset, the “cardiomegaly” classifier had the highest AUC (AUC = 1), and the “pulmonary edema” classifier had the lowest AUC (AUC = 0.97) (Supplementary Table 7). Using a predicted probability threshold of 0.5, the fine-tuned BERT model’s F1 scores were consistently within the range of the larger models’ F1 scores. For alcohol dependence and chronic pain, the fine-tuned BERT model F1 was higher than the trainer model’s F1 score (Table 1).

Additionally, results from the weak labeling experiments suggest that our CLEAR outperforms weak supervision using regular expressions, which resulted in lower average F1 scores compared to all LLMs used with CLEAR (Supplementary Table 8).

### Comparison to chunk embedding and full-note approaches

**Fig. 2:**
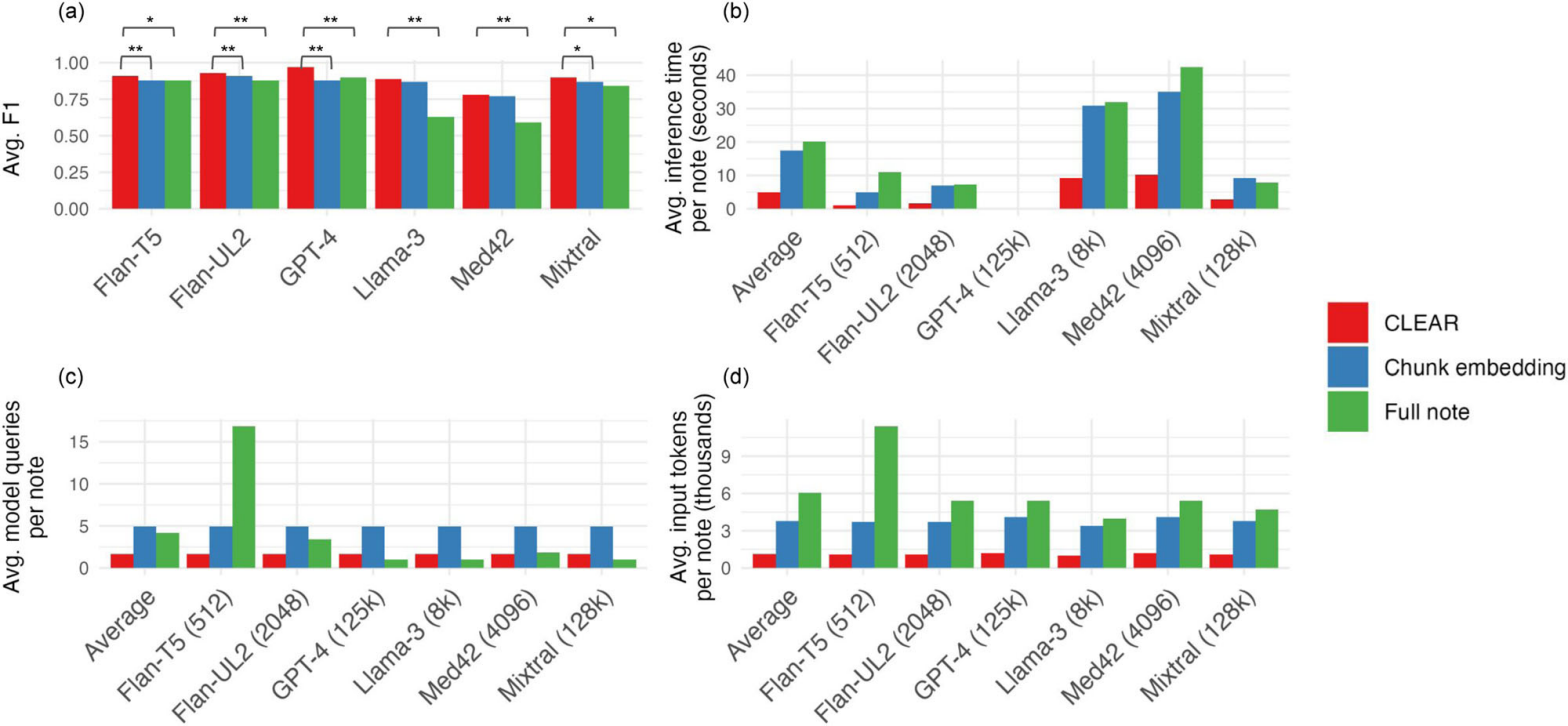
LLM information extraction comparisons on Large Token Stanford MOUD Dataset. Average F1 Score comparison between CLEAR and full-note or chunk embedding approach. F1 scores were averaged across our 13 held-out test sets. *P*-values reflect the Wilcoxon Signed-Rank Test on F1 scores across all 13 held-out test sets between CLEAR and full-note or chunk embedding comparisons (**a**). Chunk embedding top-k equals 5 in these experiments. We evaluated average inference time per note (**b**), average model queries per note (**c**), and average input tokens per note (**d**) on the Large Token Stanford MOUD Dataset across full note, chunk embeddings, and CLEAR methods for five models. Chunk embedding top-k equals 5 in these experiments. All metrics are calculated on 4xNVIDIA A100 80GB GPUs. To calculate the total tokens retrieved for GPT-4, we used Med42 as the representative tokenizer. *p < 0.05, **p < 0.01.

Across all models, chunk embedding (top-5) and full-note methods performed worse on the information extraction task compared to CLEAR (Supplementary Table 9). The performance delta was largest for GPT-4 (average F1 0.97 CLEAR vs. 0.88 chunk embedding vs. 0.90 full note) and smallest for Flan-T5 (average F1 0.91 CLEAR vs. 0.88 chunk embedding vs. 0.88 full note) (Fig. 2a, Supplementary Table 9). Increasing top-k improves chunk embedding performance, but even with *k* = 10, CLEAR outperformed chunk embedding across all models except Med42, where the chunk embedding approach outperformed CLEAR by 0.01 (F1 0.79 vs. 0.78) (Supplementary Table 10). We conducted additional experiments by increasing the CLEAR context window size from +/− 150 words to +/− 185 words, and reducing token chunks for chunk embeddings from 490 to 390. As a result, the token counts for CLEAR became larger than those for chunk embedding. We re-ran our analysis using Mixtral, and the results showed that the average F1 score for CLEAR increased by 0.01, while the average F1 score for chunk embedding decreased by 0.02 (Supplementary Table 11), compared to the original results in Supplementary Table 9.

CLEAR outperformed chunk embedding and full-note approaches on nearly all efficiency metrics. Average inference time per note ranged from 1.04 s (Flan-T5) to 10.24 s (Med42) for CLEAR; from 4.92 s (Flan-T5) to 35.07 s (Med42) for chunk embedding; and from 7.20 s (Flan-UL2) to 42.43 s (Med42) for full note. The average number of model calls per note was 1.68 for CLEAR vs. 4.94 for chunk embedding. These numbers were the same across models since all models were called once per retrieved chunk. For the full-note approach, the note was chunked according to the context window of each model. As a result, models with large input token limits—like GPT-4 (125k) and Mixtral (128k)—required fewer model calls. The average number of input tokens per note was substantially less in CLEAR compared to chunk embedding and full note. On average, CLEAR had 81% fewer input tokens than the full-note approach and 71% fewer input tokens than chunk embedding (Fig. 2b–d and Supplementary Table 12). We estimated the time required for human evaluators to extract the same information from the training data by recording how long it took our domain expert to annotate 100 notes. On average, it took 57 s for a human to annotate one variable in a clinical note, which would result in approximately 3299 h to complete the annotation of 13 variables in 16,031 notes. In comparison, CLEAR would process approximately 1.681 note chunks per note, resulting in 26,948 model calls for this same annotation task. Our fastest model takes an average time of 1.039 s per note chunk, reducing the task to 101 h, while the slowest model takes approximately 10.241 s, totaling 997 h. This represents a 96.9% efficiency gain with the fastest model and a 69.8% gain with the slowest, compared to human annotation.

We calculated ROUGE-L F-measures to test whether chunk embedding performed worse at information extraction when the retrieved text overlapped less with the text retrieved by CLEAR. When both CLEAR and chunk embedding succeeded (true positives and true negatives), the average ROUGE-L was 77%. When CLEAR succeeded, but chunk embedding failed (false positives and false negatives), ROUGE-L was also 77%, suggesting that performance differences cannot be attributed to lack of overlap in the retrieved text (*p*-value > 0.05). However, the average top-k ranks for TPs and TNs with the highest F-measure (TPs = 3.12, TNs = 4.08) were more favorable than those for FPs and FNs (FPs = 4.11, FNs = 5) (*p*-value = 0.01), indicating that the embedding similarity measure used by the chunk embedding method may not effectively prioritize the most relevant chunks (Supplementary Table 13). Overall, while both CLEAR and chunk embedding methods retrieve similarly high-yield content, CLEAR proves to be a more efficient information retrieval tool, returning relevant content in fewer chunks (4.94 average chunk embedding chunks per note vs. 1.681 average CLEAR embedding chunks per note) (Supplementary Table 12).

## Discussion

In this paper, we propose CLEAR, a RAG pipeline that retrieves note excerpts containing clinical-named entities relevant to the input query. We show that CLEAR, when used for extraction of 13 variables from clinical notes, outperformed chunk embedding and full-note approaches, achieving 3% higher F1 on average with 71% fewer input tokens, 72% faster inference time, and 66% fewer model queries. We also demonstrated that CLEAR outputs can be used to fine-tune BERT-sized models for variable extraction, resulting in performance comparable to larger models.

Our analysis suggests that CLEAR outperforms chunk embedding and full-note approaches for two main reasons. First, CLEAR retrieves shorter context segments. Prior studies have shown that longer contexts can degrade LLM performance. For example, in the FlenQA dataset, which involves three reasoning tasks, Levy et al. observed that as input length increases, model performance deteriorates regardless of whether the key information is located at the beginning, middle, or end of the input context, and that degradation occurs well before reaching the context limit of the models^42^. Similarly, Liu et al. report the “lost in the middle” phenomenon, where LLMs perform worse when key information is buried in the middle of the input context compared to being at the beginning or end^52^. They also noted that models with longer context capabilities, such as the 16k versions of GPT-3.5, did not outperform shorter context models. In our own analysis, models like Mixtral, Llama, and GPT-4, despite having context windows large enough to accommodate multiple notes, did not perform as well as CLEAR when processing the full note.

Second, we noted that the embedding model tends to rank chunks differently than CLEAR, often downranking critical chunks. This observation is consistent with our findings that chunk embedding performance improves as the number of chunks retrieved increases from 3 to 5 to 10. Note that we processed each chunk in separate model calls rather than within a single large context. Prior research supports the idea that retrieval of most similar document chunks is not always optimal. For instance, Gan et al. propose METRAG, which combines a similarity model with a utility model for retrieval, finding that their approach outperforms traditional similarity-based RAG approaches across various QA datasets.

CLEAR’s use of NER aligns with a robust precedent in RAG methodologies. A recent review of RAG approaches included 16 studies that incorporate entity recognition and entity-based reasoning in different ways for RAG^53^. For instance, NER can be employed to edit or revise generated content. In CBRKBQA, NER aids in revising results by aligning generated relations with those in the local neighborhood of the query entity within a knowledge graph^54^. Similarly, GMT-KBQA re-ranks retrieved entities and relations and conducts relation classification and entity disambiguation prior to generation^55^. Beyond content revision, several approaches use entities to extract information directly from knowledge graphs. For example, FC-KBQA, StructGPT, and KAPING retrieve relevant triplets and facts based on entity matching^49,56,57^. Xu et al. search across entities to identify relevant subgraphs in knowledge graphs for customer support issues^58^, and KnowledgeNavigator leverages NER for iterative filtering of relations to retrieve pertinent triplets from knowledge graphs^59^. Furthermore, RHO integrates entity embedding with knowledge graph embeddings to enhance dialog generation^60^. These methodologies underscore the versatility of NER in RAG, not only for retrieving information but also for structuring and refining content generation. NER can also be used to facilitate automated knowledge graph generation, suggesting that CLEAR could be used to both generate knowledge graphs^61^ and retrieve from them to improve LLM performance^47–49^.

Our study faces certain limitations. First, we restricted our evaluation to the task of clinical variable extraction. Future research should explore the performance of CLEAR on other tasks that can benefit from retrieval, including summarization, question answering, and clinical reasoning. Utilizing benchmarks like MedAlign^62^ can provide a more comprehensive evaluation of CLEAR’s capabilities across a broader range of tasks. Second, in our chunk embedding comparison, we segmented chunks based on the context window of the embedding model. While this approach is consistent with prior methods^3^, it is possible that using different-sized embedding chunks could yield similar accuracy to CLEAR. However, in our experiment, where we increased the CLEAR token size and decreased the chunk embedding token size, CLEAR still outperformed chunk embedding (see Supplementary Table 11). These findings are consistent with the data in Supplementary Table 13, where CLEAR and chunk embedding methods do not retrieve substantially different information, and increasing CLEAR’s context size does not negatively impact CLEAR’s performance. Instead, chunk embedding underperforms because the embedding similarity measures may not effectively prioritize the most relevant chunks. We believe further exploration is warranted, although a deep dive was beyond the scope of this paper. Future experiments should investigate the impact of chunk size tuning on performance. Third, our task required the information retrieval LLM process only one note chunk at a time. This can be adapted if the task requires extracting information from multiple note types. Several CLEAR note chunks from different notes can be combined into a single prompt for LLM inference, however, this was not explored in our paper. Fourth, additional prompt tuning for CLEAR steps (NER, LLM augmentation, and entity selection) is needed for full optimization, and language could have been made more consistent between the prompts used at different stages of the pipeline. Fifth, changes in data over time are inherent in medical studies. The data split we selected resulted in a higher proportion of COVID and post-COVID era notes in the Stanford MOUD testing dataset, which may contain a higher proportion of notes reflecting worsened mental health among patients^63^. Although we made efforts to check for imbalances in our training and testing datasets, these inherent differences may still exist and could impact our evaluation. Lastly, our analysis did not incorporate model quantization methods for LLM inference. Implementing model quantization could strike a balance between efficiency and performance, making it a valuable area for future research. By optimizing model configurations through quantization, we can enhance scalability and applicability in diverse contexts without compromising on performance, thereby providing more comprehensive insights into the optimal use of CLEAR in clinical information extraction.

Traditional methods of using LLM for clinical information extraction are time-consuming and cost-prohibitive. Our work introduces a more efficient RAG pipeline that identifies relevant note chunks using clinical NER before performing variable extraction, leading to a more than 70% reduction in both token usage and processing time. Importantly, these efficiencies were achieved with a slight gain in performance when compared to approaches that utilize entire documents or embed note chunks for retrieval. This work demonstrates that the application of LLMs in healthcare can be made more affordable and practical. We have validated this method in the context of variable extraction, showing its potential to transform the landscape of clinical information processing in healthcare settings.

## Methods

### Data source

We used data from two EHR-derived datasets from Stanford Hospital. The first was the Stanford Medication for Opioid Use Disorder (MOUD) cohort^6,64^. This cohort includes data from patients treated for opioid use disorder at Stanford Hospital between 2009 and 2023. Patients aged 18–89 who were prescribed buprenorphine-naloxone for more than a day were included. The cohort was split into a training and testing dataset by treatment start dates, using data up to 2020 for training and from 2021 onwards for testing. The treatment start date was used to split the data to simulate data cut-offs that would be expected in a real-world deployment of CLEAR. To evaluate the similarity between the training and testing data after the date split, we analyzed the proportion of key concept mentions (unemployment, homelessness, food insecurity, substance dependence, suicidal ideation, depression, overdose) in both sets. Using a regular expression search with synonyms for each variable (Supplementary Table 14), we found minor differences in concept mentions across the datasets (Supplementary Table 15). There were 767 patients in the training dataset with 16031 unique notes and 505 patients in the testing dataset with 12319 unique notes. Combined, the testing and training datasets had a min, median, and max token lengths of 218, 1778, and 10,981, respectively. Thirteen variables were selected for manual annotation by a board-certified addiction medicine physician due to their importance in delivering medication-assisted therapy. These variables included clinical diagnoses (depression, alcohol dependence, substance use disorder, ADHD, bipolar disorder, chronic pain, liver disease, personality disorder, PTSD, suicidal behavior, tobacco dependence) and social determinants of health (housing and employment status. All data were de-identified using the Safe Harbor method according to NIST guidelines, with clinical text undergoing additional anonymization via the TiDE algorithm^65^. Approval for the study was obtained from the Stanford University Institutional Review Board, protocol number 67423. This study was an analysis of routinely collected EHR data, and posed no additional risk to patients.

The second data source was CheXpert, a dataset of radiology reports from Stanford Hospital^66^ with programmatic labels for five well-defined clinical entities commonly found in chest x-ray reports: cardiomegaly, pulmonary edema, pleural effusion, pneumonia, and pneumothorax. These five were selected at random out of the 14 labeled observations in CheXpert. We downsampled the CheXpert dataset into a testing and training dataset by using the existing CheXpert agent’s labels to randomly sample from the larger CheXpert dataset. For the training dataset, we randomly sampled 700 notes for each of the five selected clinical entities. We only sampled notes that the CheXpert agent labeled as “present” or “negated” to upsample notes with information relevant to our retrieval task. We used a similar approach for the testing dataset, sampling 200 notes per entity, 100 of which had been labeled by CheXpert as “present” and 100 as “negated”. After selection, CheXpert labels were discarded for both datasets. In total, we had 3500 patients containing 3500 unique notes in the testing dataset, and 1000 patients containing 1000 unique notes in the training dataset. Combined, the testing and training datasets had a min, median, and max token length of 41, 189, and 1025, respectively.

To prevent data leakage, we removed testing notes for any patients whose IDs were present in the training data. This step ensured that no patient appeared in both the testing and training datasets for the Stanford MOUD and CheXpert tasks.

### Data annotation

Five board-certified physicians and one medical student collaboratively performed manual annotation of clinical variables to obtain reference labels. We randomly sampled 420 unique notes from the Stanford MOUD testing dataset to generate reference labels for 13 clinical entities outlined in Supplementary Table 16. To reduce class imbalance skewed towards negative and absent cases, we filtered the 420 unique notes using patient-level structured data (ICD-10 codes) and a regular expression search, returning notes containing any of the specified strings or from patients with at least one relevant ICD-10 code (Supplementary Table 17). Ultimately, we created 13 individual annotation datasets. The labels generated from these 13 datasets were used as our held-out test sets. 247 notes (20 from each dataset) were randomly selected for duplicate annotation to calculate inter-rater reliability (IRR). For the CheXpert test set, we generate reference labels for all 1000 notes outlined in Supplementary Table 1. 100 notes from this subset were randomly selected for duplicate annotation to calculate inter-rater reliability. For the Stanford MOUD and CheXpert datasets, labelers received specific instructions that outlined the criteria for annotating each variable. Their task involved identifying and labeling notes for the presence, absence, or uncertainty of a variable.

For each annotated variable, annotators received instructions to improve consistency. Instructions for the Stanford MOUD Dataset annotation task can be found in Supplementary Table 18. Annotators labeled positive mentions of a variable as present. Negation or absent mentions were both treated similarly. Ambiguous instances were marked as uncertain. 12 notes in the Stanford MOUD Dataset and 8 notes in CheXpert had conflicting duplicate-annotated labels for IRR. These notes were excluded from the held-out test sets.

To evaluate the sensitivity of our information retrieval pipeline, one medical student annotated a specialized dataset known as the Stanford MOUD NER Dataset. This dataset was created by randomly selecting 215 zero-shot NER input texts from the Stanford MOUD Dataset and manually extracting clinically relevant entities and concepts. Instructions for the annotation task can be found in Supplementary Table 19. We used this to evaluate the sensitivity of our information retrieval pipeline on real-world clinical datasets. After annotation, we had 450 unique clinical entities and concepts for our evaluation. Details outlining the creation of the zero-shot NER input texts can be found below.

Full details on all datasets used in this study can be found in Supplementary Fig. 1.

### Clinical entity augmented retrieval

**Fig. 3:**
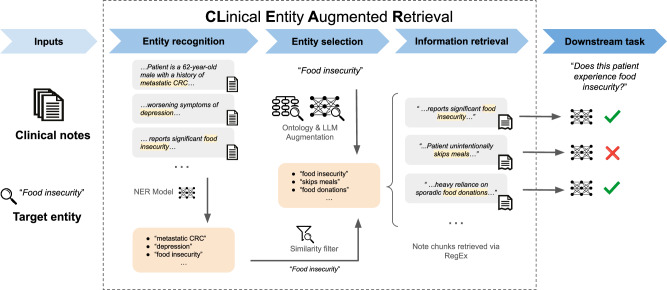
Overview of CLEAR pipeline. CLEAR requires two inputs: (1) clinical notes and (2) a target entity. Initially, our CLEAR implementation applies an NER model to the clinical notes to extract a dataset of relevant entities. These entities are then filtered using word embeddings and cosine similarity to ensure relevance to the target entity. Next, additional entities related to the target entity are identified using ontologies and LLMs. The final list of entities is used to retrieve note chunks through regular expression matches. These chunks support a downstream LLM task (clinical information extraction).

CLEAR uses NER to improve the accuracy and efficiency of clinical LLM tasks. CLEAR takes in two inputs: clinical notes and entities of interest. The pipeline begins with NER to identify all clinical entities within the notes. Next, the identified entities are filtered down to those relevant to the entities of interest. The filtered list is then augmented using ontologies and LLMs to increase sensitivity. The augmented list is fed to a target matcher that retrieves a context window surrounding each relevant entity. The retrieved-context windows can be used for downstream tasks like summarization, question answering, or information extraction. This multi-step approach is outlined below and in Fig. 3.

The first step in the CLEAR pipeline is identifying all clinical entities in the input clinical notes using a NER model. The output of this step is a list of unique clinical entities contained in the notes. We implemented the initial NER step using zero-shot NER with Flan-T5-XXL due to its specialized NER instruction tuning^67^. Most clinical NER models are domain-specific and are highly dependent on the dataset they were fine-tuned on. To minimize these limitations, we chose Flan-T5, a domain-agnostic model, for NER. The model was run on a PHI-compliant virtual machine with 8xNVIDIA L4 24GB GPUs. Given the 512 context window limit of Flan-T5, we used prompts of fewer than 20 tokens and chunked input text to under 100 tokens with a 15-token stride. We used four distinct prompts, and the output-named entities from each prompt were aggregated and de-duplicated to form the final list of entities. Illustrations of each prompt type are provided in Supplementary Fig. 2. Our approach leverages NER prompts to capture all clinically named entities; however, users have the ability to craft more focused NER prompts (e.g., “Return all named entities related to congestive heart failure”).

To evaluate the performance of our zero-shot NER approach, we used two datasets: (1) the NCBI Disease Dataset, which contains annotations for 1382 unique disease names and concepts^68^, (2) the Stanford MOUD NER Dataset, which contains annotations for 450 clinical entities and concepts. We report the sensitivity of Flan-T5-XXL in identifying these entities. Additionally, we characterize the false negatives into the following categories:

1. **Acronym recognition failures**: the model recognized either the full term or an acronym for a concept, but not both (ex: “colorectal cancer” was identified, but “crc” was missed).
2. **Morphological variance failures**: the model recognized either the pleural or singular noun version of the concept, but not both (ex: “glioblastoma” was identified, but “glioblastomas” was missed).
3. **Partial failures**: model failed to recognize the same concept in different contexts (ex: “tay sachs disease”, “tay sachs mutation”, “ashkenazi tay sachs disease”, “tay sachs disease gene” were identified, but “tay sachs” was missed).
4. **Other failures**: zero-shot NER failures that do not fall into any of the other categories (ex: “retinitis punctata albescens” was missed).

We also investigated the impact of removing the NER step on the overall performance of CLEAR. On all 13 variables in the Stanford MOUD Dataset, we ran the CLEAR pipeline with and without the initial NER step. When running CLEAR without NER, the only entities selected are those identified during entity augmentation (described below) with ontologies and LLMs. The selected entities were then used to retrieve sections of the note that were passed to GPT-4 to extract information about a variable of interest. We report the average F1 of the information extraction task.

Once the unique clinical entities from all notes are identified, the entities relevant to the input target entity are selected. These selected entities are eventually used to retrieve relevant context windows for downstream LLM tasks. First, all entities identified via NER and the target entity were embedded using Bio+Clinical BERT^69^, and those entities with a cosine similarity ≥0.85 compared to the target entity were retained. We selected a cosine similarity threshold of 0.85 after empirical testing attempting to balance the exclusion of irrelevant entities with the retention of relevant ones. The resulting entities were passed to GPT-4 with a prompt to filter the list to those most relevant to the target entity (Supplementary Fig. 3). The entity filtering step is modular, allowing users to apply Bio+Clinical BERT cosine similarity, an LLM, a human, or any combination to improve entity selection.

Next, the filtered entity list is augmented to account for entities missed during NER. Without an augmentation step, entities missed during NER could lead to incorrect context retrieval downstream. For example, if the entity “lesch nyhan” was missed by NER, and the target entity is “Lesch-Nyhan syndrome”, the downstream information retrieval might fail to retrieve sections of the note that mention “lesch nyhan”. Here, we used the UMLS ontology^70^ and GPT-4 to augment the list of entities from NER. We used the search endpoint from the UMLS API to retrieve concept names related to the target entity, and retained concept names originating from the National Library of Medicine Metathesaurus or SNOMED CT^71^. We also prompted GPT-4 to generate synonyms for the target entity (Supplementary Fig. 4).

To evaluate the impact of the entity augmentation step, we measured to what extent ontology and LLM augmentation recover entities missed in the NER step (false negatives). To evaluate the impact of the entity augmentation step, we measured how well ontology and LLM augmentation recover entities missed in the NER step (false negatives), as minimizing false negatives is crucial for downstream information retrieval. We prioritized maximizing sensitivity/recall, as false positives can be managed by the downstream LLM task, whereas false negatives result in complete loss of information. For each entity missed by the NER step, we treat a variant of the missed entity as the target entity and use the UMLS ontology and GPT-4 to generate synonyms as described above. For example, the formal name of an entity was used (“Lesch-Nyhan syndrome”) if a variant was missed (“lesch nyhan”). If the formal name was the missed term, a broader term that would encompass the formal name (“purine salvage deficiencies”) was used as the target entity. We report the proportion of entities missed during NER that were recovered through this augmentation step.

The selected entities are used to develop a regular expression tool for information retrieval. Specifically, we employed the Target Matcher provided by MedSpaCy^72^. We used the TargetRule class from the MedSpaCy NER module for identifying mentions of the selected entities within clinical notes and then pulled a context window of 150 words before and after the target entity. These retrieved-context windows are passed to an LLM for downstream inference tasks.

### Information extraction

We used the retrieved-context windows from CLEAR to extract the information (ex: is the feature present, negated/absent, or uncertain) of 13 variables in the Stanford MOUD dataset and 5 variables in the CheXpert dataset. We compared the performance of several models with different context windows on this task. These models included: Med42–70b^73^, Mixtral-8x7B-Instruct-v0.1^74^, Llama-3–70b^75^, Flan-T5-XXL^67^, Flan-UL2^76^, and GPT-4^77^. We ran GPT-4 via a secure Azure PHI-compliant instance. The other five models were run on a PHI-compliant virtual machine with 4xNVIDIA A100 80GB GPUs.

We designed prompts that included synthetic in-context examples generated by GPT-4^78^. We included five examples for each entity, covering all possible labels that the LLM was to discern: “0” for entity negated/absence, “1” for presence, and “2” for uncertainty. We selected 5-shot prompting based on its demonstrated performance gains in prior work^36^. To mitigate any potential issues with the context window limitations of each model, we kept the synthetic data points under 100 tokens. We kept each example under 100 tokens to ensure they provide meaningful insights into the task, improving LLM instruction following without consuming excessive tokens. In comparing our methods to the traditional approach, also known as the full-note method, we accounted for the increase in token length due to our prompting strategies, ensuring the models’ token limits were not exceeded. An example of our LLM information extraction prompt is provided in Supplementary Fig. 5.

For each model, we report classification metrics (sensitivity, specificity, NPV, PPV, F1) comparing the LLM information extraction labels to human-annotated labels.

### Weak labeling

We compared CLEAR to a weak labeling approach. Weak supervision was used to label the 2000 CheXpert notes in the training set for our five CheXpert entities of interest. Labeling functions are rough heuristics used to programmatically generate weak labels from unlabeled data. We manually reviewed the 250 notes in the training set and created labeling functions using keyword matching and regular expressions from a list of synonyms created by a domain expert and supplemented by GPT-4 using the prompt in Supplementary Fig. 4. The final list of synonyms can be found in Supplementary Table 20. For example, a labeling function might label a note as having cardiomegaly if it contains the strings “enlarged cardiac silhouette,” “enlarged heart,” or “ventricular hypertrophy,” and abstain otherwise. The labeling functions for each entity were used to train a model that combines the outputs of multiple labeling functions for a given entity, leveraging their collective knowledge and handling their conflicts^79^.

### Model distillation

We investigated whether the output of CLEAR could be used to fine-tune a smaller language model to perform the information extraction task. We used the output of CLEAR to fine-tune BERT models to perform a binary classification task (present vs. negated/absent) for each of the variables in the Stanford MOUD and CheXpert datasets. We omitted the “uncertain” class due to small sample sizes in certain fine-tuning datasets (Supplementary Table 21). We fine-tuned Bio+Clinical BERT, which was initialized from BioBERT and trained on all MIMIC notes^69^. For each variable, we selected the best performing LLM, excluding GPT-4 on the information extraction task to weakly label the fine-tuning dataset (Supplementary Table 21). We excluded GPT-4 since OpenAI terms of use prohibit using GPT-4 outputs to develop competitor models^80^. The fine-tuning dataset for each variable consisted of every note chunk containing an entity of interest (inputs) and label generated by an LLM (label). We removed note chunks that contained >10% overlapping words, resulting in less than 2% of note chunks being filtered out.

The fine-tuning datasets for each variable were divided into a 70% training set and a 30% validation set. Hyperparameters were tuned using 10-fold cross-validation on 70% of the training data to maximize the area under the receiver operating curve (AUC). We selected a range of variables for each hyperparameter and performed a grid search to find the best hyperparameter configurations. Our grid search included learning rate (5e-5, 3e-5, 2e-5), batch size (8, 16, 32), number of training epochs (4, 5, 10), and weight decay (0.01, 0.05, 0.1). The final models were fine-tuned on 100% of the fine-tuning dataset. Performance metrics for the fine-tuned classifier were generated using the held-out test sets. To prevent data leakage, we removed testing notes for any patients whose IDs were present in the fine-tuning data (training and validation datasets). This ensured that no patient appeared in both the held-out test set and fine-tuning datasets for the Stanford and CheXpert variables.

### Comparison to chunk embedding and full-note approaches

To quantify the impact of retrieving text around entities, we compared CLEAR to a RAG pipeline leveraging note chunk embeddings and a naive approach that retrieves the full note. We filtered the test sets down to longer notes to focus this comparison on notes that approached or exceeded models’ input context window. Specifically, we select the 50% longest notes in the Stanford MOUD Dataset.

For the chunk embedding RAG pipeline, we used the BAAI Generalized Embeddings (BGE) model as our embedding model and cosine similarity as the retriever (Supplementary Fig. 6). BGE is a high-performance embedding model known for its accuracy on retrieval benchmarks^81^. We first segmented all patient notes into chunks of 490 tokens with a stride of 128 tokens, given BGE’s maximum context window of 512. We then generated embeddings for each chunk as well as every target entity and its definition and stored them in an embedding database. To select the most relevant note chunks for our information extraction task, we perform cosine similarity to measure the alignment of each note chunk against the target entity’s definition embedding. We retrieved the top-*k* (where *k* = 3, 5, 10) note chunks based on cosine similarity scores. For notes with fewer than k chunks, we retrieved all chunks. The retrieved chunks were passed to an LLM for the information extraction task. For notes with multiple chunks, we aggregated the LLM labels from these chunks to generate a final label for the note.

For the full-note approach, we chunked notes based on each model’s context window limit, using a stride of 128 tokens^4^ (Supplementary Fig. 7). We passed each chunk to an LLM for the information extraction task and aggregated the labels from these segments to produce a final label for each note.

For all LLMs, we compare the performance of CLEAR, chunk embeddings, and the full-note approach on the information extraction tasks as well as on three metrics related to inference efficiency. For the information extraction tasks, we report the average inference time per note, average model queries per note, and average tokens retrieved per note. We do not report inference time for GPT-4 since it was run using a proprietary API. We used the Wilcoxon Signed-Rank Test to compare the differences between the three methods^82^.

We tested two hypotheses regarding performance differences between CLEAR and chunk embedding. First, we hypothesized that chunk embeddings would perform worse than CLEAR when the retrieved chunks overlapped less with the chunks retrieved by CLEAR. To test this, we calculated ROUGE-L F-measure—a measure of the longest common subsequence between two strings—on the chunks retrieved by CLEAR and chunk embedding, treating the CLEAR chunks as the reference. We report ROUGE-L for cases where both CLEAR and chunk embeddings succeeded (true negatives or true positives), and for cases where CLEAR succeeded but chunk embeddings failed (false negatives and false positives). Second, we hypothesized that chunk embeddings would perform worse than CLEAR when the parts of the note retrieved by CLEAR were ranked lower by the chunk embedding model. To do this, we calculated the chunk embedding model ranking of the chunk that overlapped most with the CLEAR chunk (as measured by ROUGE-L).

### Model summary

For NER, we relied on Flan-T5-XXL. LLM Augmentation used GPT-4. Our entity selection cosine similarity model was Bio+ClinicalBERT, and the entity filtering LLM was GPT-4. Information extraction was tested on six models: Med42–70b, Mixtral-8x7B-Instruct-v0.1, Llama-3–70b, Flan-T5-XXL, Flan-UL2, and GPT-4. The chunk embedding model was BAAI Generalized Embeddings Large English v1.5, and for model distillation, we fine-tuned Bio+ClinicalBERT (Supplementary Table 22). Model usage parameters for NER and information extraction are reported in Supplementary Table 23.

## Supplementary information

Supplemental figures and tables

## Data Availability

The Stanford MOUD Cohort Dataset used in this study contains identifiable protected health information and, therefore, cannot be shared publicly. Stanford University investigators with appropriate IRB approval can contact the authors directly regarding data access.

## Code availability

The code used to run CLEAR and reproduce results can be found at https://github.com/iv-lop/clear.
