# Supplementary material for: Clinical entity augmented retrieval for clinical information extraction
Source: NPJ Digit Med. 2025 Jan 19;8:45. doi: 10.1038/s41746-024-01377-1 (PMC11743751; doi:10.1038/s41746-024-01377-1)
Supplement: Supplementary file 1 — Supplemental figures and tables [file 41746_2024_1377_MOESM1_ESM.pdf]

# Supplementary material

| Supplementary Table 1: Zero-shot NER evaluation on NCBI Disease and Stanford MOUD Datasets | | | | |
|---|---|---|---|---|
| Dataset | NCBI Disease Dataset (n = 1,382) | | Stanford MOUD NER Dataset (n = 450) | |
| Method | Zero-shot NER | Zero-shot NER + LLM/Ontology augmentation | Zero-shot NER | Zero-shot NER + LLM/Ontology augmentation |
| Sensitivity | 0.96 | 0.99 | 0.98 | 1 |

| Supplementary Table 2: Zero-shot NER prompt specific evaluation on the Stanford MOUD Dataset | |
|---|---|
| Prompt | Prompt Sensitivity |
| catchAll | 0.96 |
| medSocial | 0.47 |
| clinical | 0.91 |
| medical | 0.88 |
| Combined | 0.98 |

| | Supplementary Table 3: Information Retrieval Error Classification and Augmentation Experiment Results | | | |
|---|---|---|---|---|
| | NCBI Disease Dataset | | Stanford MOUD NER Dataset | |
| Error Type | Total Errors | Augmentation Rescue Type (%) | Total Errors | Augmentation Rescue Type (%) |
| Acronym Recognition | 14 | Ontology (100%) | 1 | Ontology (100%) |
| Morphological Variance | 10 | LLM (80%) | 0 | NA |
| Partials Failure | 10 | LLM (100%) | 1 | LLM (100%) |
| Other Failure | 15 | LLM (100%) | 8 | LLM (100%) |

| Supplementary Table 4: CLEAR Information Retrieval Ablation F1 Scores on Stanford MOUD Dataset | | | | |
|---|---|---|---|---|
| Entity | NER | Ontology Augmentation | LLM Augmentation | Ontology+LLM Augmentation |
| Depression | 0.97 | 0.83 | 0.83 | 0.90 |
| Alcohol Dependence | 0.91 | 0.55 | 0.41 | 0.59 |
| Substance Use Disorder | 0.91 | 0.28 | 0.46 | 0.51 |
| Unhoused | 0.97 | 0.54 | 0.97 | 0.97 |
| Tobacco Dependence | 0.99 | 0.62 | 0.08 | 0.65 |
| Personality Disorder | 1.00 | 1.00 | 0.38 | 1.00 |
| Bipolar Disorder | 1.00 | 0.74 | 0.57 | 0.82 |
| PTSD | 1.00 | 0.98 | 0.84 | 0.98 |
| ADHD | 0.97 | 0.97 | 0.72 | 0.97 |
| Suicidal Behavior | 0.99 | 0.97 | 0.95 | 0.98 |
| Liver Disease | 0.99 | 0.84 | 0.65 | 0.97 |
| Chronic Pain | 0.95 | 0.89 | 0.88 | 0.91 |
| Unemployment | 1.00 | 0.74 | 1.00 | 1.00 |
| Average | 0.97 | 0.76 | 0.67 | 0.86 |
| We reported F1 scores as well as the average F1 score across all 13 variables using GPT-4 with CLEAR leveraging only NER, Ontology, LLM, or Ontology+LLM Augmentation for information retrieval on the Stanford MOUD Dataset. | | | | |

| Supplementary Table 5: NER Key Terms Missing From Ontology+LLM Augmentations of Stanford MOUD Dataset | | |
|---|---|---|
| Feature | Unique NER Entity | Appearance Frequency |
| Depression | insomnia | 49 |
| | depressed mood | 16 |
| | suicidal ideation | 16 |
| | suicidal behavior | 11 |
| | suicidal thoughts | 6 |
| | psychiatric hospitalization | 2 |
| Alcohol Dependence | alcohol use | 81 |
| | withdrawal | 72 |
| | etoh | 25 |

| | | |
|---|---|---|
| | alcohol withdrawal | 11 |
| | abuse screening | 3 |
| | addict | 2 |
| | history of substance abuse | 2 |
| | last drink | 1 |
| Substance Use Disorder | opioid | 64 |
| | smoking | 58 |
| | withdrawal | 55 |
| | methadone | 49 |
| | opiate | 45 |
| | opioids | 45 |
| | tobacco use | 35 |
| | nicotine | 32 |
| | opioid use | 30 |
| | addiction medicine | 28 |
| | ambien | 28 |
| | opiates | 28 |
| | substance use | 28 |
| | ketamine | 24 |
| | polysubstance use | 23 |
| | alcohol | 20 |
| | detox | 19 |
| | drinking | 18 |
| | heroin use | 18 |
| | drug use | 15 |
| | polysubstance abuse | 13 |
| | heroin | 12 |
| | benzodiazepines | 11 |
| | heroin abuse | 11 |
| | marijuana | 10 |
| | cocaine | 7 |
| | codeine | 7 |
| | smokeless tobacco | 7 |

| | | |
|---|---|---|
| | alcohol withdrawal | 5 |
| | heroin abuse disorder | 5 |
| | methamphetamine | 4 |
| | alcohol use | 3 |
| | history of substance abuse | 2 |
| | methadone injection | 2 |
| | addict | 1 |
| | drug seeking behavior | 1 |
| | opiate use disorder | 1 |
| | tobacco abuse | 1 |
| Tobacco Dependence | addiction | 79 |
| | substance use | 51 |
| | substance abuse | 39 |
| | smoking status | 37 |
| | smoking | 28 |
| | nicotine gum | 7 |
| | nicotine patch | 5 |
| | smokeless tobacco | 5 |
| | quit smoking | 4 |
| | every day smoker | 1 |
| | quitting | 1 |
| Bipolar Disorder | olanzapine | 27 |
| | lithium | 11 |
| | lamotrigine | 9 |
| | valproate | 3 |
| | bipolar d/o | 2 |
| | bi polar disorder | 1 |
| Post Traumatic Stress Disorder | psychosocial stressors | 4 |
| Suicidal Behavior | self mutilation | 1 |
| Liver Disease | hepatic steatosis | 16 |
| | hepatomegaly | 9 |
| | hepatology | 7 |
| | acute liver failure | 4 |

| | | |
|---|---|---|
| | liver lesion | 3 |
| | acute liver injury | 2 |
| | portal hypertension | 2 |
| Chronic Pain | pain management | 94 |
| | pain medication | 34 |
| | pain management plan | 16 |
| | chronic pain on suboxone | 8 |
| | chronic headaches | 4 |
| | chronic pain issues | 4 |
| | continuous use of opioids | 1 |

Supplementary Table 6: CLEAR, Full Note, and Chunk Embedding F1 Scores on Large Token Stanford MOUD Dataset

| Model | Method | Depression | Alcohol Dependence | Substance Use Disorder | Unhoused | Tobacco Dependence | Personality Disorder | Bipolar Disorder | PTSD | ADHD | Suicidal Behavior | Liver Disease | Chronic Pain | Unemployment | Average F1 Score |
|---|---|---|---|---|---|---|---|---|---|---|---|---|---|---|---|
| Flan-T5 | CLEAR | 0.76 | 0.93 | 0.92 | 0.94 | 0.92 | 0.77 | 0.97 | 0.98 | 0.95 | 1.00 | 0.76 | 0.98 | 1.00 | 0.91 |
| Flan-UL2 | CLEAR | 0.84 | 0.81 | 0.90 | 0.94 | 0.98 | 0.91 | 0.93 | 0.98 | 0.95 | 0.98 | 0.96 | 0.98 | 0.95 | 0.93 |
| GPT-4 | CLEAR | 0.93 | 0.90 | 0.89 | 1.00 | 0.98 | 1.00 | 1.00 | 1.00 | 0.96 | 1.00 | 0.98 | 0.96 | 1.00 | 0.97 |
| Med42 | CLEAR | 0.56 | 0.83 | 0.81 | 0.92 | 0.81 | 0.50 | 0.96 | 0.87 | 0.67 | 0.78 | 0.54 | 0.95 | 0.94 | 0.78 |
| Llama-3 | CLEAR | 0.84 | 0.77 | 0.86 | 1.00 | 0.90 | 0.91 | 0.88 | 0.98 | 0.91 | 0.93 | 0.71 | 0.99 | 0.89 | 0.89 |
| Mixtral | CLEAR | 0.86 | 0.73 | 0.96 | 0.94 | 0.89 | 0.89 | 0.91 | 0.96 | 0.86 | 0.98 | 0.93 | 0.95 | 0.90 | 0.90 |
| Flan-T5 | Full Note | 0.76 | 0.87 | 0.87 | 0.89 | 0.94 | 0.71 | 0.93 | 0.90 | 0.90 | 0.97 | 0.83 | 0.96 | 0.96 | 0.88 |
| Flan-UL2 | Full Note | 0.68 | 0.77 | 0.86 | 0.88 | 0.96 | 0.83 | 0.90 | 0.96 | 0.95 | 0.98 | 0.91 | 0.96 | 0.78 | 0.88 |
| GPT-4 | Full Note | 0.86 | 0.86 | 0.92 | 1.00 | 0.90 | 0.57 | 0.90 | 1.00 | 0.92 | 1.00 | 0.95 | 0.96 | 0.86 | 0.90 |
| Med42 | Full Note | 0.51 | 0.63 | 0.74 | 0.75 | 0.26 | 0.50 | 0.85 | 0.63 | 0.55 | 0.65 | 0.43 | 0.68 | 0.44 | 0.59 |
| Llama-3 | Full Note | 0.69 | 0.62 | 0.67 | 0.62 | 0.41 | 0.50 | 0.78 | 0.91 | 0.78 | 0.72 | 0.44 | 0.83 | 0.22 | 0.63 |
| Mixtral | Full Note | 0.78 | 0.77 | 0.91 | 0.89 | 0.77 | 1.00 | 0.94 | 0.95 | 0.36 | 0.94 | 0.78 | 0.90 | 0.89 | 0.84 |
| Flan-T5 | Chunk Embedding | 0.75 | 0.84 | 0.87 | 0.94 | 0.94 | 0.71 | 0.93 | 0.98 | 0.84 | 1.00 | 0.74 | 0.95 | 0.91 | 0.88 |
| Flan-UL2 | Chunk Embedding | 0.81 | 0.81 | 0.88 | 0.94 | 0.96 | 0.77 | 0.90 | 0.96 | 0.95 | 1.00 | 0.93 | 0.96 | 0.91 | 0.91 |
| GPT-4 | Chunk Embedding | 0.87 | 0.65 | 0.86 | 0.89 | 0.94 | 0.53 | 0.93 | 1.00 | 0.87 | 1.00 | 0.98 | 0.96 | 0.90 | 0.88 |
| Med42 | Chunk Embedding | 0.56 | 0.74 | 0.80 | 0.86 | 0.81 | 0.60 | 1.00 | 0.91 | 0.78 | 0.87 | 0.49 | 0.95 | 0.67 | 0.77 |
| Llama-3 | Chunk Embedding | 0.81 | 0.73 | 0.84 | 0.93 | 0.88 | 0.86 | 0.93 | 0.96 | 0.86 | 1.00 | 0.67 | 0.98 | 0.84 | 0.87 |
| Mixtral | Chunk Embedding | 0.79 | 0.69 | 0.88 | 0.94 | 0.83 | 0.83 | 0.91 | 0.90 | 0.73 | 1.00 | 0.93 | 0.96 | 0.91 | 0.87 |

We reported F1 scores as well as the average F1 score across all 13 variables for all 6 models using CLEAR, full note, or chunk embedding for information extraction.

Supplementary Table 7: Fine-tuned Classifier Performance

| Dataset | Classifier | Sens | Spec | PPV | NPV | AUC | F1 |
|---|---|---|---|---|---|---|---|
| Stanford MOUD Dataset | Alcohol dependence | 0.94 | 0.94 | 0.89 | 0.97 | 0.97 | 0.91 |
| | ADHD | 0.88 | 0.97 | 0.94 | 0.94 | 0.91 | 0.91 |
| | Bipolar disorder | 0.95 | 0.68 | 0.81 | 0.91 | 0.92 | 0.87 |
| | Chronic pain | 1 | 0.31 | 0.88 | 1 | 0.89 | 0.94 |
| | Unhoused | 1 | 0.88 | 0.82 | 1 | 1 | 0.90 |
| | Liver disease | 0.97 | 0.83 | 0.94 | 0.9 | 0.98 | 0.95 |
| | Depression | 1 | 0.44 | 0.81 | 1 | 0.89 | 0.90 |
| | Personality disorder | 0.78 | 0.97 | 0.93 | 0.9 | 0.97 | 0.85 |
| | PTSD | 0.64 | 0.95 | 0.97 | 0.53 | 0.93 | 0.77 |
| | Substance use disorder | 0.8 | 0.91 | 0.95 | 0.69 | 0.9 | 0.87 |
| | Suicidal behavior | 0.92 | 0.42 | 0.86 | 0.57 | 0.83 | 0.89 |
| | Tobacco dependence | 1 | 0.94 | 0.97 | 1 | 1 | 0.98 |
| | Unemployment | 1 | 0.83 | 0.96 | 1 | 0.99 | 0.98 |
| CheXpert Dataset | Cardiomegaly | 0.97 | 0.95 | 0.94 | 0.98 | 0.99 | 0.95 |
| | Pleural effusion | 0.99 | 0.94 | 0.95 | 0.99 | 1 | 0.97 |
| | Pulmonary Edema | 0.84 | 0.98 | 0.95 | 0.94 | 0.96 | 0.89 |
| | Pneumonia | 0.89 | 1 | 1 | 0.92 | 0.99 | 0.94 |
| | Pneumothorax | 0.99 | 0.93 | 0.93 | 0.99 | 0.99 | 0.96 |

We used CLEAR to generate fine-tuning datasets that were used to fine-tune Bio+Clinical BERT for classification. The results in this table are fine-tuned Bio+Clinical BERT performance on the Stanford MOUD and CheXpert held-out test sets.

| Supplementary Table 8: Weak supervision experiment results for CheXpert Entities. | | | | | |
|---|---|---|---|---|---|
| Entity | Sensitivity | Specificity | PPV | NPV | F1 Score |
| Cardiomegaly | 0.90 | 0.97 | 0.96 | 0.92 | 0.92 |
| Pleural effusion | 0.98 | 0.73 | 0.80 | 0.97 | 0.88 |
| Pulmonary Edema | 1.00 | 0.29 | 0.55 | 1.00 | 0.71 |
| Pneumonia | 0.95 | 0.60 | 0.49 | 0.97 | 0.65 |
| Pneumothorax | 1.00 | 0.26 | 0.50 | 1.00 | 0.67 |
| Average | 0.97 | 0.57 | 0.66 | 0.97 | 0.77 |

| Supplementary Table 9: Average F1 Score Method Comparisons on Large Token Stanford MOUD Dataset | | | | | |
|---|---|---|---|---|---|
| Model | Full Note | Chunk Embedding* | CLEAR | CLEAR:Full Note Comparison† | CLEAR:Chunk Embedding* Comparison† |
| Flan-T5 | 0.88 | 0.88 | 0.91 | 0.0239 | 0.0044 |
| Flan-UL2 | 0.88 | 0.91 | 0.93 | 0.0019 | 0.0054 |
| GPT-4 | 0.90 | 0.88 | 0.97 | 0.0064 | 0.0046 |
| Med42 | 0.59 | 0.77 | 0.78 | 0.0013 | 0.6082 |
| Llama-3 | 0.63 | 0.87 | 0.89 | 0.0001 | 0.0732 |
| Mixtral | 0.84 | 0.87 | 0.90 | 0.0341 | 0.0227 |

F1 score averaged across 13 Stanford MOUD Dataset held-out test sets. P-values reflect Wilcoxon Signed-Rank Test on F1 scores across all 13 held-out test sets between CLEAR and full note or CLEAR and chunk embedding methods comparisons.

* Chunk embedding top-k equals 5
† Statistical test: Wilcoxon Signed-Rank Test

| Supplementary Table 10: Chunk Embedding Top-3,5,10 and CLEAR Average LLM F1 Scores for Stanford Dataset | | | | |
|---|---|---|---|---|
| Model | Chunk Embedding Top-3 | Chunk Embedding Top-5 | Chunk Embedding Top-10 | CLEAR |
| Flan-T5 | 0.84 | 0.88 | 0.89 | 0.91 |
| Flan-UL2 | 0.86 | 0.91 | 0.90 | 0.93 |
| GPT-4 | 0.86 | 0.88 | 0.87 | 0.97 |
| Med42 | 0.71 | 0.77 | 0.79 | 0.78 |
| Llama-3 | 0.82 | 0.87 | 0.87 | 0.89 |
| Mixtral | 0.85 | 0.87 | 0.87 | 0.90 |

F1 score averages across 13 Stanford MOUD Dataset held-out test sets for CLEAR and Chunk Embedding using 3 different top-k values (3, 5, and 10).

| Supplementary Table 11: Information extraction with average CLEAR chunks larger than Chunk Embedding chunks. We report F1 score for all 13 Stanford MOUD variables as well as the average. | | | | | | | | | | | | | | | | |
|---|---|---|---|---|---|---|---|---|---|---|---|---|---|---|---|---|
| Model | Method | Average Token Count | Depression | Alcohol Dependence | Substance Use Disorder | Unhoused | Tobacco Dependence | Personality Disorder | Bipolar Disorder | PTSD | ADHD | Suicidal Behavior | Liver Disease | Chronic Pain | Unemployment | Average F1 Score |
| Mixtral | CLEAR | 710 | 0.91 | 0.75 | 0.92 | 0.89 | 0.94 | 0.92 | 0.91 | 0.96 | 0.95 | 0.98 | 0.93 | 0.95 | 0.87 | 0.91 |
| Mixtral | Chunk Embedding | 675 | 0.80 | 0.65 | 0.88 | 0.94 | 0.80 | 0.71 | 0.88 | 0.92 | 0.75 | 1.00 | 0.93 | 0.96 | 0.78 | 0.85 |

| | Supplementary Table 12: LLM Information Extraction Efficiency† on Large Token Stanford MOUD Dataset | | | | | | | | |
|---|---|---|---|---|---|---|---|---|---|
| | Inference time per note (seconds) | | | Model queries per note | | | Total input tokens per note (thousands)‡ | | |
| Model (input token limit) | Full Note | Chunk Embedding* | CLEAR | Full Note | Chunk Embedding* | CLEAR | Full Note | Chunk Embedding* | CLEAR |
| Flan-T5 (512) | 11.004 | 4.925 | 1.039 | 16.831 | 4.940 | 1.681 | 11.4k | 3.7k | 1.1k |
| Flan-UL2 (2048) | 7.205 | 6.997 | 1.612 | 3.411 | | | 5.4k | 3.7k | 1.1k |
| GPT-4 (125k) | - | - | - | 1.000 | | | 5.4k | 4.1k | 1.2k |
| Mixtral (128k) | 7.850 | 9.126 | 2.757 | 1.000 | | | 4.7k | 3.8k | 1.1k |
| Med42 (4096) | 42.428 | 35.066 | 10.241 | 1.819 | | | 5.4k | 4.1k | 1.2k |
| Llama-3 (8k) | 31.899 | 30.944 | 9.125 | 1.031 | | | 4.0k | 3.4k | 1k |
| Average | 20.0772 | 17.4116 | 4.9548 | 4.182 | 4.94 | 1.681 | 6.1k | 3.8k | 1.1k |

We evaluated LLM information extraction efficiency on the Large Token Stanford MOUD Dataset across full note, chunk embedding, and CLEAR methods for five models.

* Chunk embedding top-k equals 5
† All metrics are calculated on 4xNVIDIA A100 80GB GPUs
‡ To calculate total tokens retrieved for GPT-4, we used Med42 as the representative tokenizer

| | Supplementary Table 13: Average maximum ROUGE-L F-measure and average chunk rank (out of 5) for maximum ROUGE-L F-measure of chunk embeddings with CLEAR as reference | |
|---|---|---|
| | Average chunk rank for Maximum ROUGE-L F-Measure | Average Maximum ROUGE-L F-Measure |
| Missed Case Analysis | | |
| Chunk embedding FP and CLEAR TP | 4.11 | 0.77 |
| Chunk embedding FN and CLEAR TN | 5 | 0.77 |
| Total | 4.43* | 0.77† |
| Correct Case Analysis | | |
| Chunk embedding TP and CLEAR TP | 3.12 | 0.78 |
| Chunk embedding TN and CLEAR TN | 4.08 | 0.7 |
| Total | 3.31* | 0.77† |
| *Average chunk rank p-value = 0.01 | | |
| †Average maximum ROUGE-L F-Measure p-value = 0.82 | | |
| For every TP and TN CLEAR chunk, we report the average chunk rank and ROUGE-L F-measure for the chunk embedding that had the highest F-measure with CLEAR chunks as the reference. TP, TN, FP, FN represent true positive, true negative, false positive, and false negative, respectively. P-values were calculated using T-test. | | |

| Supplementary Table 14: Synonyms used to represent a concept for regular expression matching. | |
|---|---|
| Concept | Synonyms |
| Unemployment | 'labor force nonparticipant', 'without work', 'inactive', 'not employed', 'seeking work', 'on benefits', 'disemployed', 'jobless', 'career break', 'unemployed', 'out-of-work', 'out of employment', 'idle', 'out of work', 'without a job', 'on assistance', 'on welfare', 'unoccupied', 'without occupation', 'out of a job', 'nonworking', 'non-employed', 'on unemployment', 'laid off', 'unemployment benefits recipient', 'unengaged', 'nonworker', 'not working', 'without employment', 'workless', 'not in employment', 'on the dole', 'unemployed individual', 'work-seeking', 'seeking employment', 'benefit recipient', 'economically inactive', 'between jobs', 'underemployed', 'joblessness', 'unemployment' |
| Unhoused | 'homeless status', 'transient', 'street dweller', 'without permanent residence', 'temporarily unsheltered', 'residence-less', 'on the streets', 'living on the street', 'lacking stable housing', 'shelterless', 'housing insecure', 'residential instability', 'under-housed', 'unsheltered', 'homeless', 'roofless', 'homeless individual', 'dispossessed', 'homeless life', 'urban camping', 'sidewalk inhabitant', 'chronically homeless', 'without a fixed abode', 'sleeping rough', 'homeless experience', 'living rough', 'vulnerable to homelessness', 'displaced', 'transient population', 'residentially challenged', 'non-residential', 'street-bound', 'urban homeless', 'homeless population', 'public space dweller', 'homelessness', 'homeless condition', 'pavement dwellers', 'nomadic', 'street living', 'without housing', 'housing instability', 'vagrant', 'homeless circumstances', 'itinerant', 'vagrancy', 'street homelessness', 'rough sleeper', 'inadequately housed', 'unhoused', 'street-involved', 'living without shelter', 'houseless', 'street residents', 'without shelter', 'homeless situation', 'outdoor living', 'unaccommodated', 'unhomed', 'people without homes' |
| Food insecurity | 'food shortage', 'nutrient deficiency', 'food deficit', 'food deprivation', 'dietary insufficiency', 'scarce food resources', 'limited food availability', 'food insecurity', 'inadequate dietary intake', 'undernutrition', 'starvation', 'malnutrition', 'food instability', 'limited nutritional resources', 'dietary scarcity', 'dietary deprivation', 'food poverty', 'meal insecurity', 'food insufficiency', 'caloric deficiency', 'hunger issues', 'hunger', 'nutritional deficiency', |

| | |
|---|---|
| | 'inadequate food resources', 'subsistence crisis', 'food scarcity', 'nutritional insecurity', 'poor food access', 'food inadequacy', 'inadequate food supply', 'limited food access', 'nutrient scarcity', 'insufficient nutrition', 'chronic undernourishment', 'nutritional shortfall', 'food access disparity' |
| Substance dependence | 'psychoactive substance abuse', 'illicit drug use', 'harmful drug use', 'illegal drug use', 'recreational drug use', 'chemical dependency', 'addiction', 'substance abuse', 'prescription drug abuse', 'alcoholism', 'pharmaceutical abuse', 'cocaine use disorder', 'alcohol use disorder', 'substance use disorder', 'medication use disorder', 'harmful substance use', 'substance-related disorders', 'alcohol abuse', 'risky substance use', 'amphetamine use disorder', 'amphetamine abuse', 'problematic drug use', 'opioid use disorder', 'drug addiction', 'non-medical drug use', 'sud', 'substance misuse', 'drug abuse', 'cannabis abuse', 'opioid abuse', 'drug misuse', 'cannabis use disorder', 'cocaine abuse', 'chemical abuse', 'substance dependence', 'drug dependence', 'narcotic abuse', 'intoxicant abuse', 'problematic substance use', 'substance addiction', 'psychoactive substance use disorder' |
| Suicidal ideation | 'self-injurious behavior', 'suicidal impulses', 'suicidal intent', 'self-wounding', 'suicidal expression', 'suicidal gestures', 'suicidal tendencies', 'suicidality', 'attempted suicide', 'suicidal behavior', 'suicide', 'suicide attempt', 'volitional self-harm', 'self-inflicted injury', 'deliberate self-harm', 'suicide plan', 'auto-aggression', 'suicidal planning', 'suicide threat', 'suicide proneness', 'self-inflicted harm', 'life-threatening behavior', 'self-directed violence', 'self-destructive acts', 'suicide completion', 'suicide crisis', 'self-inflicted violence', 'suicide contemplation', 'suicide risk', 'suicidal crisis', 'lethal self-harm', 'self-injury', 'self-harm', 'self-poisoning', 'suicide gesture', 'suicidal attempts', 'fatal self-harm', 'suicide behavior', 'suicidal action', 'suicidal feelings', 'self-inflicted death', 'suicidal ideation', 'self-destructive behavior', 'intentional self-harm', 'suicidal acts', 'self-suicide', 'suicide ideation', 'self-aggression', 'act of self-harm', 'self-mutilation', 'suicidal actions', 'suicidal thoughts' |
| Depression | 'chronic depression', 'depressed', 'dysthymia', 'anhedonia', 'existential depression', 'single episode depression', 'minor depression', 'premenstrual dysphoric disorder', 'cognitive depression', 'affective disorder', 'long-term |

| | |
|---|---|
| | depression', 'refractory depression', 'major depression', 'emotional dysregulation', 'secondary depression', 'mild depression', 'neurotic depression', 'persistent mood disorder', 'persistent depressive disorder', 'major depressive disorder', 'depressive disorder not otherwise specified', 'depression', 'depressive illness', 'low mood', 'seasonal affective disorder', 'major depressive episode', 'bipolar depression', 'unipolar depression', 'postpartum depression', 'chronic mood disorder', 'double depression', 'reactive depression', 'moderate depression', 'melancholic depression', 'endogenous depression', 'depressive condition', 'profound depression', 'high-functioning depression', 'atypical depression', 'psychotic depression', 'depressive symptoms', 'psychological depression', 'functional depression', 'somatization in depression', 'situational depression', 'treatment-resistant depression', 'depressive mood', 'depressive episode', 'mood disorder', 'recurrent depression', 'adjustment disorder with depressed mood', 'masked depression', 'severe depression', 'melancholia', 'mixed depression', 'vascular depression', 'depressive affect', 'mood dysregulation', 'clinical depression', 'depressive state', 'non-specific depression', 'subsyndromal depression', 'dysphoria' |
| Overdose | 'opiate overdose case', 'opioid overdose reaction', 'acute opioid toxicity', 'excessive opioid dosage', 'hydromorphone overdose', 'narcotic overdose event', 'overdose on opioids', 'opiate overconsumption', 'opioid ingestion complication', 'accidental opiate ingestion', 'opiate dose toxicity', 'opioid over administration', 'opiate poisoning', 'pain pill overdose', 'suboxone toxicity', 'prescription opioid overdose', 'opiate cns depression', 'naloxone treatment', 'overintake of opioids', 'narcotic poisoning onset', 'narcotic overconsumption', 'heroin overdose', 'opiate poisoning event', 'narcotic overdose complication', 'opioid intoxication episode', 'opioid lethal ingestion', 'narcotic toxicity symptoms', 'acute narcotic poisoning', 'opiate overdose presentation', 'morphine overdose', 'opioid overdose event', 'opiate induced coma', 'opiate toxicity', 'overconsumption of opiates', 'hydrocodone intoxication', 'excessive opiate ingestion', 'narcotic related intoxication', 'opioid od', 'narcotic related overdose', 'acute opioid overdose', 'acute narcotic overdose', 'narcotic toxic exposure', 'methadone overdose', 'accidental opioid poisoning', 'tramadol toxicity', |

| | |
|---|---|
| | 'narcotic overdose', 'opioid overdose onset', 'opioid intoxication symptoms', 'buprenorphine overdose', 'opioid related toxicity', 'narcotic toxic event', 'overactive opioid intake', 'opiate overexposure', 'fatal opioid intake', 'respiratory failure from opioids', 'painkiller overdose', 'opioid induced respiratory depression', 'prescription opiate toxicity', 'opiate central depression', 'narcotic overdose symptoms', 'overuse of opioids', 'opioid reversal', 'oxycodone overdose', 'opioid poisoning', 'opioid overuse', 'lethal opiate exposure', 'vicodin intoxication', 'acute opiate intoxication', 'opioid toxicity', 'narcotic toxic episode', 'narcotic intoxication', 'tapentadol intoxication', 'carfentanil poisoning', 'percocet overdose', 'opiate sedation overdose', 'heroin poisoning', 'opiate excess', 'codeine poisoning', 'opioid overdose episode', 'lethal opioid dose', 'opiate related overdose', 'opioid excess ingestion', 'opioid toxic exposure', 'narcotic poisoning', 'opiate intoxication', 'fentanyl toxicity', 'opioid over intake', 'opiate overdose', 'opiate toxic reaction', 'opioid ingestion excess', 'opiate overdose event', 'opiate induced overdose', 'opioid complication', 'opiate exposure toxicity' |

Supplementary Table 15: Proportion of concept mentions via synonym regular expression matching for Stanford MOUD training and testing datasets.

| Dataset | Unemployment | Unhoused | Food insecurity | Substance dependence | Suicidal ideation | Depression | Overdose |
|---|---|---|---|---|---|---|---|
| Train | 0.0155 | 0.0268 | 0.0278 | 0.113 | 0.0328 | 0.118 | 0.00293 |
| Test | 0.0187 | 0.0416 | 0.0274 | 0.106 | 0.0233 | 0.085 | 0.00471 |

| Supplementary Table 16: Held-out test sets manually labeled by clinicians | | | | |
|---|---|---|---|---|
| Variable | Present | Negated/Absent | Uncertain | Total Notes |
| Stanford MOUD | | | | |
| Alcohol dependence | 25 | 43 | 2 | 70 |
| ADHD | 17 | 30 | 1 | 48 |
| Bipolar disorder | 30 | 25 | 13 | 68 |
| Chronic pain | 73 | 9 | 8 | 90 |
| Unhoused | 14 | 24 | 0 | 38 |
| Liver disease | 45 | 18 | 9 | 72 |
| Depression | 62 | 28 | 4 | 94 |
| Personality disorder | 16 | 35 | 0 | 51 |
| PTSD | 41 | 18 | 1 | 60 |
| Substance use disorder | 39 | 19 | 6 | 64 |
| Suicidal behavior | 53 | 13 | 1 | 67 |
| Tobacco dependence | 47 | 24 | 4 | 75 |
| Unemployment | 22 | 5 | 0 | 27 |
| CheXpert | | | | |
| Cardiomegaly | 76 | 100 | 24 | 200 |
| Pleural effusion | 97 | 80 | 23 | 200 |
| Pulmonary Edema | 94 | 105 | 1 | 200 |
| Pneumonia | 44 | 108 | 48 | 200 |
| Pneumothorax | 84 | 110 | 6 | 200 |

| Supplementary Table 17: ICD-10 codes and string search elements used to filter the Stanford MOUD cohort for patient notes with a higher likelihood of containing one of the 13 concepts. | | |
|---|---|---|
| Concept | ICD-10 Codes | String Search |
| Depression | F31.31, F31.32, F31.4, F31.5, F31.6x, F31.9, F32.0, F32.1, F32.2, F32.3, F32.4, F32.5, F32.8, F32.9, F32.9, F33.0, F33.1, F33.2, F33.3, F33.41, F33.42, F33.8, F33.9, F34.1, F34.8, F34.9, F38.0, F38.1, F38.8, F39, F41.2, F43.2, F43.2, F99 | ['chronic depression', 'depressed', 'dysthymia', 'anhedonia', 'existential depression', 'single episode depression', 'minor depression', 'premenstrual dysphoric disorder', 'cognitive depression', 'affective disorder', 'long-term depression', 'refractory depression', 'major depression', 'emotional dysregulation', 'secondary depression', 'mild depression', 'neurotic depression', 'persistent mood disorder', 'persistent depressive disorder', 'major depressive disorder', 'depressive disorder not otherwise specified', 'depression', 'depressive illness', 'low mood', 'seasonal affective disorder', 'major depressive episode', 'bipolar depression', 'unipolar depression', 'postpartum depression', 'chronic mood disorder', 'double depression', 'reactive depression', 'moderate depression', 'melancholic depression', 'endogenous depression', 'depressive condition', 'profound depression', 'high-functioning depression', 'atypical depression', 'psychotic depression', 'depressive symptoms', 'psychological depression', 'functional depression', 'somatization in depression', 'situational depression', 'treatment-resistant depression', 'depressive mood', 'depressive episode', 'mood disorder', 'recurrent depression', 'adjustment disorder with depressed mood', 'masked depression', 'severe depression', 'melancholia', 'mixed depression', 'vascular depression', 'depressive affect', 'mood dysregulation', 'clinical depression', 'depressive state', 'non-specific depression', 'subsyndromal depression', 'dysphoria'] |
| Alcohol Dependence | F10.x, G62.1, G31.2, G72.1, I42.6, K29.2, K70.0-K70.4, K70.9, K85.2, K86.0, Q86.0, P04.3, I85, K22.6, K74.0-K74.2, K74.6, K76.0, K76.7, K76.9, K76.6, X45, Y15, X65, Z71.4x | ['alcoholic', 'dipsomania', 'alcohol misuse', 'drinking problem', 'alcohol addiction', 'excessive drinking', 'alcoholism', 'heavy drinking', 'etoh abuse', 'etoh addiction', 'hazardous drinking', 'alcohol dependency syndrome', 'alcohol dependency', 'ethanol dependence', 'uncontrolled drinking', 'alcohol use disorder', 'alcoholic disease', 'ethanol addiction', 'alcohol abuse', 'aud', 'risky drinking', 'etoh dependence', 'unhealthy alcohol use', 'alcohol-related disorder', 'chronic alcoholism', 'addiction to alcohol', 'problem drinking', 'ethanol abuse', 'alcohol dependence', 'binge drinking', 'alcohol excess', 'alcohol overuse'] |
| Substance Use Disorder | F10.x, F11.x, F12.x, F13.x, F14.x, F15.x, F16.x, F17.x, F18.x, F19.x | ['psychoactive substance abuse', 'illicit drug use', 'harmful drug use', 'illegal drug use', 'recreational drug use', 'chemical dependency', 'addiction', 'substance abuse', 'prescription drug abuse', 'alcoholism', 'pharmaceutical abuse', |

| | | |
|---|---|---|
| | | 'cocaine use disorder', 'alcohol use disorder', 'substance use disorder', 'medication use disorder', 'harmful substance use', 'substance-related disorders', 'alcohol abuse', 'risky substance use', 'amphetamine use disorder', 'amphetamine abuse', 'problematic drug use', 'opioid use disorder', 'drug addiction', 'non-medical drug use', 'sud', 'substance misuse', 'drug abuse', 'cannabis abuse', 'opioid abuse', 'drug misuse', 'cannabis use disorder', 'cocaine abuse', 'chemical abuse', 'substance dependence', 'drug dependence', 'narcotic abuse', 'intoxicant abuse', 'problematic substance use', 'substance addiction', 'psychoactive substance use disorder'] |
| Unhoused | Z59.x | ['homeless status', 'transient', 'street dweller', 'without permanent residence', 'temporarily unsheltered', 'residence-less', 'on the streets', 'living on the street', 'lacking stable housing', 'shelterless', 'housing insecure', 'residential instability', 'under-housed', 'unsheltered', 'homeless', 'roofless', 'homeless individual', 'dispossessed', 'homeless life', 'urban camping', 'sidewalk inhabitant', 'chronically homeless', 'without a fixed abode', 'sleeping rough', 'homeless experience', 'living rough', 'vulnerable to homelessness', 'displaced', 'transient population', 'residentially challenged', 'non-residential', 'street-bound', 'urban homeless', 'homeless population', 'public space dweller', 'homelessness', 'homeless condition', 'pavement dwellers', 'nomadic', 'street living', 'without housing', 'housing instability', 'vagrant', 'homeless circumstances', 'itinerant', 'vagrancy', 'street homelessness', 'rough sleeper', 'inadequately housed', 'unhoused', 'street-involved', 'living without shelter', 'houseless', 'street residents', 'without shelter', 'homeless situation', 'outdoor living', 'unaccommodated', 'unhomed', 'people without homes'] |
| Tobacco Dependence | F17.x, O99.33x, P04.2, P96.81, T65.2x, Z57.31, Z71.6, Z72.0, Z77.2x, Z87.8 | ['cigarette habit', 'tobacco abuse', 'electronic cigarette dependence', 'compelled to smoke', 'smoking addiction', 'tobacco habit', 'tobacco consumption syndrome', 'nicotine patch reliance', 'tobacco cessation struggle', 'cigarette dependence', 'dependent on smoking', 'nicotine habituation', 'nicotine driven', 'cigar dependence', 'tobacco use disorder', 'addicted to smokeless tobacco', 'cigarette tethered', 'nicotine gum dependence', 'continuous smoker', 'nicotine cycle', 'smoking habituation', 'tobacco user', 'chronic smoker', 'regular cigarette use', 'tobacco product dependent', 'hooked on tobacco', 'inhaler smoker dependent', 'smokeless tobacco dependence', 'long term smoker', 'smoking reliant', 'hooked on nicotine', 'tobacco craving', 'nicotine stick dependence', 'regular tobacco user', 'heavy |

| | | |
|---|---|---|
| | | smoker', "can't resist smoking", 'nicotine withdrawal symptoms', 'nicotine dependence profile', 'nicotine use disorder', 'tobacco use compulsion', 'nicotine abused', 'tobacco compulsive behavior', 'cigarette craving', 'frequent smoker', 'pipe smoker dependence', 'constant smoking', 'nicotine chewer', 'inability to stop smoking', 'tobacco hooked', 'nicotine enslaved', 'tobacco consumption habit', 'tobacco stick user', 'tobacco intake dependency', 'tobacco inhaler', 'persistent smoking', 'tobacco compulsive use', 'daily tobacco intake', 'nicotine lozenge reliance', 'tobacco chewer dependence', 'nicotine craving', 'tobacco obsessed', 'nicotine tethered', 'tobacco use syndrome', 'tobacco habituated', 'nicotine compulsive', 'persistent tobacco craving', 'vape dependence', 'tobacco enslaved', 'constant craving for tobacco', 'tobacco tethered', 'snuff tobacco dependence', 'nicotine user', 'addicted to tobacco products', 'nicotine fixated', 'compulsive smoking', 'dependence on cigars', 'tobacco consumption disorder', 'tobacco chewer habit', 'nicotine snuff dependence', 'tobacco fixated', 'nicotine reliant', 'smoker status', 'tobacco user profile', 'chain smoker', 'nicotine addiction', 'nicotine pouch use', 'tobacco use reliant', 'smoke dependent', 'tobacco reliant behavior', 'smoking dependency syndrome', 'tobacco bound', 'dependent on nicotine', 'cigarette use disorder', 'nicotine consumption disorder', 'addicted to cigarettes', 'nicotine obsessed', 'tobacco withdrawal', 'addiction to smoke', "can't quit smoking", 'nicotine dependence', 'unable to quit tobacco', 'chronic tobacco use', 'struggling with tobacco quitting'] |
| Personality disorder | F60.x | ['narcissistic personality disorder', 'personality disorder', 'schizoid personality disorder', 'character disorder', 'interpersonal relational disorders', 'fixed personality traits', 'singular personality disorders', 'borderline personality disorder', 'cluster b personality disorders', 'adaptive dysfunction', 'distorted personality patterns', 'anxious personality disorders', 'impulsive personality disorder', 'adaptive personality traits', 'unorthodox personality patterns', 'histrionic personality disorder', 'persistent personality patterns', 'idiosyncratic personality disorders', 'dramatic personality disorders', 'nonconformist personality traits', 'unusual personality disorders', 'obsessive-compulsive personality disorder', 'unconventional personality disorders', 'interpersonal dysfunction disorders', 'affective dysregulation disorders', 'atypical personality disorders', 'irregular personality patterns', 'idiosyncratic character traits', 'affective personality disorders', 'eccentric personality disorders', 'behavioral character disorder', |

| | | |
|---|---|---|
| | | 'abnormal personality patterns', 'emotional dysregulation disorder', 'deviant personality traits', 'pathological personality traits', 'personality maladjustment', 'avoidant personality disorder', 'chronic personality disorders', 'aberrant personality traits', 'personality pathology', 'psychodynamic personality disorders', 'social dysfunction disorders', 'enduring personality traits', 'personality disturbance', 'rigid personality patterns', 'behavioral personality disorders', 'complex personality dysfunction', 'personality dysfunction', 'maladaptive personality traits', 'non-standard personality traits', 'anomalous personality disorders', 'schizotypal personality disorder', 'paranoid personality disorder', 'ego-dystonic personality disorders', 'temperamental disorders', 'cluster c personality disorders', 'exceptional personality disorders', 'inflexible personality traits', 'dependent personality disorder', 'character pathology', 'ego-syntonic personality disorders', 'peculiar personality patterns', 'dysfunctional personality traits', 'personality spectrum disorders', 'divergent personality patterns', 'antisocial personality disorder', 'psychological personality disorders', 'cluster a personality disorders'] |
| Bipolar disorder | F31.x | ['mania', 'manic', 'hypomanic', 'hypomania', 'bipolar diagnosis', 'bipolar mood swings', 'bipolar mood disturbance', 'hypo manic state', 'manic depression', 'euphoric mania', 'bipolar affective disorder', 'elevated mood', 'bipolar progression', 'manic depressive illness', 'sub threshold bipolar', 'bpad', 'pre manic state', 'bipolar related', 'manic onset', 'cyclic mood swings', 'recurrent depressive episodes', 'hypomania', 'bipolar flare up', 'bipolar dysregulation', 'bipolar mood fluctuation', 'bipolarity', 'bp ii', 'manic state', 'bipolar break', 'chronic bipolar disorder', 'manic dysthymia', 'bipolar type 2', 'bipolar subtype', 'bipolar relapse', 'bipolar related disorders', 'bipolar with anxious distress', 'mood dysregulation', 'bipolar ii disorder', 'bipolar manic', 'rapid cycling', 'bd', 'bipolar disorder', 'bipolar tendencies', 'bipolar remission', 'bipolar episode', 'manic symptomatology', 'bipolar depression', 'bipolar mixed', 'bipolar switch', 'bipolar presentation', 'cycling mood disorder', 'bipolar maintenance phase', 'mood disorder', 'bipolar ii', 'mixed bipolar state', 'bipolar type 1', 'cyclothymic disorder', 'dysphoric mania', 'ultra rapid cycling', 'bipolar disorder with psychotic features', 'mood elevation', 'bipolar i disorder', 'bipolar i', 'bipolar shift', 'manic episode', 'mixed episode', 'cyclothymia', 'mood stabilization', 'manic psychosis', 'post bipolar depression', 'bp i', 'manic mood', 'mood swings', 'bipolar depressive phase', 'post manic depression', 'manic phase', 'ultra ultra rapid cycling', 'bipolar |

| | | |
|---|---|---|
| | | with seasonal pattern', 'bipolar with mixed features', 'hypomanic episode', 'bipolar transition', 'manic polarity', 'inter episode bipolar', 'early onset bipolar', 'bipolar with rapid cycling', 'treatment resistant bipolar', 'bipolar spectrum disorder', 'bipolar cycle', 'acute bipolar', 'bipolar mood episodes', 'bipolar nos (not otherwise specified)', 'refractory bipolar'] |
| PTSD | F43.x | ['traumatic war neurosis', 'psychological trauma', 'combat fatigue syndrome', 'adjustment reaction to adult life', 'psychotraumatic syndrome', 'operational fatigue', 'chronic post-traumatic stress', 'delayed stress syndrome', 'emotional shock', 'secondary traumatic stress', 'combat stress reaction', 'post traumatic stress syndrome', 'post-combat stress', 'occupational stress injury', 'psychological stress disorder', 'battle fatigue', 'shell shock', 'trauma-related psychological disorder', 'stress aftermath', 'traumatic neurosis', 'post-traumatic emotional disorder', "veteran's neurosis", "soldier's heart", 'military stress syndrome', 'post-traumatic stress injury', 'ptsd', 'trauma- and stressor-related disorder', 'shock syndrome', 'war neurosis', 'war stress injury', 'stress response syndrome', 'traumatic war experience reaction', 'stress-induced psychosis', 'survivor syndrome', 'acute stress disorder', 'stress collapse', 'operational stress injury', 'traumatic stress disorder', 'acute stress reaction', 'post traumatic stress disorder'] |
| ADHD | F90.x | ['psychiatric attention deficit', 'developmental impulsivity disorder', 'neuropsychiatric developmental disorder', 'behavioral impulsivity', 'disruptive behavior disorder', 'childhood hyperactivity', 'hyperactivity-impulsivity disorder', 'adolescent adhd', 'psychomotor impulsivity', 'cognitive impairment disorder', 'attention deficiency', 'add', 'attention-deficit syndrome', 'hyperkinetic syndrome', 'attentional hyperactivity', 'executive dysfunction', 'neurobehavioral disorder', 'hyperactivity', 'adult adhd', 'childhood impulsivity disorder', 'inattention', 'attention deficit hyperactivity disorder', 'childhood onset adhd', 'impulsivity', 'psychosocial disorder', 'hyperactive-impulsive syndrome', 'conduct disorder', 'attentional deficit', 'behavioral disorder', 'attention inconsistency disorder', 'focus deficit disorder', 'behavioral regulation disorder', 'impulsive hyperactivity', 'attention deficit disorder', 'adhd', 'concentration deficit disorder', 'executive function disorder', 'attention dysregulation', 'hyperkinetic disorder', 'neurodevelopmental impairment', 'hyperactive disorder'] |
| Liver disease | K70.x, K71.x, K72.x, K73.x, K74.x, K75.x, | ['hepatitis e', 'cholestasis from hepatitis', |

| | | |
|---|---|---|
| | K76.x, K77.x | 'hepatitis related fatigue', 'hbv dna positive', 'hepatitis serology positive', 'hepatic ultrasound for hepatitis', 'hepatitis vaccination history', 'chronic persistent hepatitis', 'hepatitis related dark urine', 'igg anti hbc positive', 'hepatitis related neuropathy', 'drug induced hepatic', 'fulminant hepatic failure', 'liver function tests for hepatitis', 'chronic liver inflammation', 'liver inflammation', 'hepatitis related edema', 'liver panel indicating hepatitis', 'hbv', 'necroinflammatory liver disease', 'hepatitis related pruritus', 'chronic hepatic', 'chronic active hepatitis', 'igm anti hav positive', 'hav igm', 'acute hepatic', 'hepatic fibrosis', 'liver biopsy showing hepatitis', 'hepatitis coinfection', 'envelope antibody', 'hepatitis related anorexia', 'hepatic virus serology', 'hepatitis related abdominal pain', 'autoimmune hepatic', 'alcoholic hepatitis', 'non a non b hepatitis', 'anti hbc', 'hepatitis', 'hepatitis related leukopenia', 'hepatic inflammation', 'viremia from hepatitis', 'hepatic steatosis from hepatitis', 'hepatic lesion from hepatitis', 'hepatitis immunoglobulin treatment', 'alt elevation from hepatitis', 'hepatitis related jaundice', 'hepatitis related pale stools', 'hepatitis related vomiting', 'hepatitis related fever', 'hepatitis related thrombocytopenia', 'hepatic viral', 'idiopathic hepatitis', 'hepatitis related myalgia', 'hcv genotyping', 'envelope antigen', 'toxic hepatitis', 'viral hepatitis', 'hepatitis a', 'hcv', 'liver disease', 'hepatitis related nausea', 'hepatitis related splenomegaly', 'fatty liver from hepatitis', 'hepatitis related ascites', 'hev', 'core antibody', 'hdv', 'liver cirrhosis from hepatitis', 'hepatotropic', 'hepatitis associated anemia', 'surface antigen', 'hepatitis related arthralgia', 'hav', 'delta hepatitis', 'viral hepatic', 'hepatitis related spider angiomas', 'cirrhosis', 'hepatitis d', 'non viral hepatitis', 'hepatitis related malaise', 'hepatitis related hepatomegaly', 'hepatitis related rash', 'post hepatitis syndrome', 'ast elevation from hepatitis', 'due to hepatitis', 'liver inflammation markers', 'autoantibodies in hepatitis', 'liver infection', 'surface antibody', 'hbsag', 'hepatitis b', 'hepatitis c', 'viral liver', 'hepatic encephalopathy'] |
| Suicidal behavior | R45.2, R45.84, R45.85x, R45.88, R45.89, T14.91, Z91.51, Z91.52, F32, F60.3, X71-X83 | ['self-injurious behavior', 'suicidal impulses', 'suicidal intent', 'self-wounding', 'suicidal expression', 'suicidal gestures', 'suicidal tendencies', 'suicidality', 'attempted suicide', 'suicidal behavior', 'suicide', 'suicide attempt', 'volitional self-harm', 'self-inflicted injury', 'deliberate self-harm', 'suicide plan', 'auto-aggression', 'suicidal planning', 'suicide threat', 'suicide proneness', 'self-inflicted harm', 'life-threatening behavior', 'self-directed violence', 'self-destructive acts', 'suicide completion', 'suicide crisis', 'self-inflicted |

| | | |
|---|---|---|
| | | violence', 'suicide contemplation', 'suicide risk', 'suicidal crisis', 'lethal self-harm', 'self-injury', 'self-harm', 'self-poisoning', 'suicide gesture', 'suicidal attempts', 'fatal self-harm', 'suicide behavior', 'suicidal action', 'suicidal feelings', 'self-inflicted death', 'suicidal ideation', 'self-destructive behavior', 'intentional self-harm', 'suicidal acts', 'self-suicide', 'suicide ideation', 'self-aggression', 'act of self-harm', 'self-mutilation', 'suicidal actions', 'suicidal thoughts'] |
| Chronic pain | G89.x, R50-R69, M54.1x, M54.2, M54.3x, M54.4x, M54.5x, M54.6x, M79.x | ['nagging pain', 'chronic radiating pain', 'ongoing pain', 'long lasting pain', 'chronic cramping', 'prolonged pain', 'chronic mechanical pain', 'chronic post surgical pain', 'chronic idiopathic pain', 'chronic pain flare', 'chronic visceral pain', 'permanent pain', 'recurring pain', 'unceasing pain', 'persistent soreness', 'chronic sharp pain', 'chronic pangs', 'ever present pain', 'continual discomfort', 'chronic degenerative pain', 'non acute pain', 'chronic stinging', 'chronic throbbing', 'chronic psychogenic pain', 'chronic nerve pain', 'persistent ache', 'chronic neuralgia', 'chronic migraine pain', 'consistent discomfort', 'ceaseless pain', 'chronic regional pain', 'chronic discomfort', 'chronic surface pain', 'chronic burning', 'long term pain', 'long standing pain', 'chronic musculoskeletal pain', 'chronic phantom pain', 'chronic pelvic pain', 'everlasting pain', 'extended pain', 'chronic deep pain', 'prolonged soreness', 'relentless pain', 'chronic arthritic pain', 'chronic refractory pain', 'unending pain', 'chronic tenderness', 'recurrent pain', 'chronic intractable pain', 'chronic hurt', 'persistent pain', 'chronic back pain', 'chronic post traumatic pain', 'prolonged discomfort', 'consistent pain', 'chronic pain syndrome', 'chronic referred pain', 'steady pain', 'chronic stabbing', 'perpetual pain', 'chronic soreness', 'chronic dullness', 'chronic fibromyalgia', 'lasting ache', 'constant pain', 'chronic pain disorder', 'chronic joint pain', 'incessant ache', 'persistent throbbing', 'chronic nociceptive pain', 'chronic widespread pain', 'chronic dull ache', 'enduring pain', 'continued pain', 'continual pain', 'constant ache', 'chronic muscle pain', 'chronic affliction', 'chronic neuropathic pain', 'chronic non malignant pain', 'chronic localized pain', 'chronic inflammation pain', 'chronic ache', 'undying pain', 'continuing pain', 'persistent discomfort', 'chronic trigeminal pain', 'chronic inflammatory pain', 'chronic pain', 'chronic breakthrough pain', 'chronic myofascial pain', 'chronic headache'] |
| Unemployment | Z56.x | ['labor force nonparticipant', 'without work', 'inactive', 'not employed', 'seeking work', 'on benefits', 'disemployed', 'jobless', 'career break', 'unemployed', 'out-of-work', 'out of |

| | | |
|---|---|---|
| | | employment', 'idle', 'out of work', 'without a job', 'on assistance', 'on welfare', 'unoccupied', 'without occupation', 'out of a job', 'nonworking', 'non-employed', 'on unemployment', 'laid off', 'unemployment benefits recipient', 'unengaged', 'nonworker', 'not working', 'without employment', 'workless', 'not in employment', 'on the dole', 'unemployed individual', 'work-seeking', 'seeking employment', 'benefit recipient', 'economically inactive', 'between jobs', 'underemployed', 'joblessness', 'unemployment'] |

| Supplementary Table 18: Stanford MOUD held-out test set annotation instructions | |
|---|---|
| Concept | Instructions |
| Depression | For this task, we are labeling notes as ‘positive’ (with a 1) if they suggest the patient has had a history of depression. Here, ‘depression’ is a broad term that can fall under a larger umbrella for a patient having a history of ‘depression’. We are not looking to fulfill any diagnostic clinical criteria, we want you to use your clinical intuition based on what you would label this note. We are choosing to go with a clinical diagnosis realizing that formal diagnosis criteria aren’t readily available in a patient’s notes. Here are some examples of terms that fall under our umbrella categorization of a patient having a history of ‘depression’: 'chronic depression', 'depressed', 'mild depression', 'clinical depression', 'major depressive disorder', 'postpartum depression', etc…). If the note does not suggest the patient has a history of the concept (concept is absent or negated) then label the note as a 0. If you are uncertain then label the note as a 2.<br><br>Here are some examples of 0, 1, and 2 labels:<br><br>#####<br>text: the patient was diagnosed with major depression in 2019<br>label: 1<br><br>#####<br>text: the patient has clinical depression<br>label: 1<br><br>#####<br>text: it was uncertain whether the patient experienced depression<br>label: 2<br><br>#####<br>text: patient has a history of depression<br>label: 1<br><br>#####<br>text: there is no evidence of mdd but the patient has a history of glioblastoma and crc<br>label: 0 |
| Alcohol Dependence | For this task, we are labeling notes as ‘positive’ (with a 1) if they suggest the patient has had a history of alcohol dependence. Here, ‘alcohol dependence’ is a broad term that can fall under a larger umbrella for a patient having a history of ‘alcohol dependence’. We are not looking to fulfill any diagnostic clinical criteria, we want you to use your clinical intuition based on what you would label this note. We are choosing to go with a clinical diagnosis realizing that formal diagnosis criteria aren’t readily available in a patient’s notes. Here are some examples of terms that fall under our umbrella categorization of a patient having a history of ‘alcohol dependence’: 'alcoholic', 'alcohol misuse', 'drinking problem', 'alcohol addiction', 'alcohol dependency', 'ethanol dependence', 'uncontrolled drinking', 'alcohol use disorder', 'alcohol overuse', etc…). If the note does not suggest the patient has a history of the concept (concept is absent or negated) then label the note as a 0. If you are uncertain then label the note as a 2.<br><br>Here are some examples of 0, 1, and 2 labels:<br><br>##### |

| | |
|---|---|
| | text: the patient was diagnosed with alcohol use disorder in 2019<br>label: 1<br><br>#####<br>text: the patient has alcohol dependence<br>label: 1<br><br>#####<br>text: it was uncertain whether the patient has alcohol addiction<br>label: 2<br><br>#####<br>text: patient has a history of alcoholism<br>label: 1<br><br>#####<br>text: there is no evidence of aud but the patient has a history of glioblastoma and crc<br>label: 0 |
| Substance Use Disorder | For this task, we are labeling notes as ‘positive’ (with a 1) if they suggest the patient has had a history of substance use disorder. Here, substance use disorder is a broad term that can fall under a larger umbrella for a patient having a history of substance use disorder. We are not looking to fulfill any diagnostic clinical criteria, we want you to use your clinical intuition based on what you would label this note. We are choosing to go with a clinical diagnosis realizing that formal diagnosis criteria aren’t readily available in a patient’s notes. Here are some examples of terms that fall under our umbrella categorization of a patient having a history of substance use disorder: 'substance abuse', 'prescription drug abuse', 'alcoholism', 'pharmaceutical abuse', 'cocaine use disorder', etc…). If the note does not suggest the patient has a history of the concept (concept is absent or negated) then label the note as a 0. If you are uncertain then label the note as a 2.<br><br>Here are some examples of 0, 1, and 2 labels:<br><br>#####<br>text: the patient was diagnosed with substance use disorder in 2019<br>label: 1<br><br>#####<br>text: the patient has a drug addiction<br>label: 1<br><br>#####<br>text: it was uncertain whether the patient has sud<br>label: 2<br><br>#####<br>text: patient has a history of alcohol use disorder<br>label: 1<br><br>#####<br>text: there is no evidence of active opioid addiction but the patient has a history of glioblastoma and crc<br>label: 0 |

| | |
|---|---|
| Unhoused | For this task, we are labeling notes as ‘positive’ (with a 1) if they suggest the patient has had a history of being unhoused. Here, unhoused is a broad term that can fall under a larger umbrella for a patient having a history of being unhoused. We are not looking to fulfill any diagnostic clinical criteria, we want you to use your clinical intuition based on what you would label this note. We are choosing to go with a clinical diagnosis realizing that formal diagnosis criteria aren’t readily available in a patient’s notes. Here are some examples of terms that fall under our umbrella categorization of a patient having a history of being unhoused: 'homeless status', 'unhoused', 'without permanent residence', 'temporarily unsheltered', etc…). If the note does not suggest the patient has a history of the concept (concept is absent or negated) then label the note as a 0. If you are uncertain then label the note as a 2.<br><br>Here are some examples of 0, 1, and 2 labels:<br><br>#####<br>text: the patient has been homeless since 2019<br>label: 1<br><br>#####<br>text: the patient is currently unhoused<br>label: 1<br><br>#####<br>text: it is uncertain whether the patient is houseless<br>label: 2<br><br>#####<br>text: patient relies on transitional housing for shelter<br>label: 1<br><br>#####<br>text: the patient has stable housing but is struggling financially<br>label: 0 |
| Tobacco Dependence | For this task, we are labeling notes as ‘positive’ (with a 1) if they suggest the patient has had a history of tobacco dependence. Here, tobacco dependence is a broad term that can fall under a larger umbrella for a patient having a history of tobacco dependence. We are not looking to fulfill any diagnostic clinical criteria, we want you to use your clinical intuition based on what you would label this note. We are choosing to go with a clinical diagnosis realizing that formal diagnosis criteria aren’t readily available in a patient’s notes. Here are some examples of terms that fall under our umbrella categorization of a patient having a history of tobacco dependence: 'tobacco abuse', 'electronic cigarette dependence', 'compelled to smoke', 'smoking addiction', etc…). If the note does not suggest the patient has a history of the concept (concept is absent or negated) then label the note as a 0. If you are uncertain then label the note as a 2.<br><br>Here are some examples of 0, 1, and 2 labels:<br><br>#####<br>text: the patient was diagnosed with nicotine dependence in 2019<br>label: 1<br><br>#####<br>text: the patient has a tobacco addiction |

| | |
|---|---|
| | label: 1<br><br>#####<br>text: it is uncertain whether the patient has a nicotine addiction<br>label: 2<br><br>#####<br>text: patient has a history of tobacco dependence<br>label: 1<br><br>#####<br>text: there is no evidence of smoking addiction but the patient has a history of glioblastoma and crc<br>label: 0 |
| Personality disorder | For this task, we are labeling notes as 'positive' (with a 1) if they suggest the patient has had a history of personality disorder. Here, personality disorder is a broad term that can fall under a larger umbrella for a patient having a history of personality disorder. We are not looking to fulfill any diagnostic clinical criteria, we want you to use your clinical intuition based on what you would label this note. We are choosing to go with a clinical diagnosis realizing that formal diagnosis criteria aren't readily available in a patient's notes. Here are some examples of terms that fall under our umbrella categorization of a patient having a history of personality disorder: 'narcissistic personality disorder', 'personality disorder', 'schizoid personality disorder', etc…). If the note does not suggest the patient has a history of the concept (concept is absent or negated) then label the note as a 0. If you are uncertain then label the note as a 2.<br><br>Here are some examples of 0, 1, and 2 labels:<br><br>#####<br>text: the patient was diagnosed with paranoid personality disorder in 2019<br>label: 1<br><br>#####<br>text: the patient has borderline personality disorder<br>label: 1<br><br>#####<br>text: it is uncertain whether the patient has dependent personality disorder<br>label: 2<br><br>#####<br>text: patient has a history of ocpd<br>label: 1<br><br>#####<br>text: there is no evidence of schizoid personality disorder but the patient has a history of glioblastoma and crc<br>label: 0 |
| Bipolar disorder | For this task, we are labeling notes as 'positive' (with a 1) if they suggest the patient has had a history of bipolar disorder. Here, bipolar disorder is a broad term that can fall under a larger umbrella for a patient having a history of bipolar disorder. We are not looking to fulfill any diagnostic clinical criteria, we want you to use your clinical intuition based on what you would label this note. We are choosing to go with a clinical |

| | |
|---|---|
| | diagnosis realizing that formal diagnosis criteria aren't readily available in a patient's notes. Here are some examples of terms that fall under our umbrella categorization of a patient having a history of bipolar disorder: 'bp ii', 'bipolar disorder with psychotic features', 'bipolar i disorder', 'manic depression', etc…). If the note does not suggest the patient has a history of the concept (concept is absent or negated) then label the note as a 0. If you are uncertain then label the note as a 2.<br><br>Here are some examples of 0, 1, and 2 labels:<br><br>#####<br>text: the patient was diagnosed with bipolar disorder in 2019<br>label: 1<br><br>#####<br>text: the patient has manic depression<br>label: 1<br><br>#####<br>text: it is uncertain whether the patient has bipolar I<br>label: 2<br><br>#####<br>text: patient has a history of bipolar affective disorder<br>label: 1<br><br>#####<br>text: there is no evidence of bipolar II disorder but the patient has a history of glioblastoma and crc<br>label: 0 |
| PTSD | For this task, we are labeling notes as 'positive' (with a 1) if they suggest the patient has had a history of PTSD. Here, PTSD is a broad term that can fall under a larger umbrella for a patient having a history of PTSD. We are not looking to fulfill any diagnostic clinical criteria, we want you to use your clinical intuition based on what you would label this note. We are choosing to go with a clinical diagnosis realizing that formal diagnosis criteria aren't readily available in a patient's notes. Here are some examples of terms that fall under our umbrella categorization of a patient having a history of PTSD: 'post-traumatic stress', 'post traumatic stress disorder', etc…). If the note does not suggest the patient has a history of the concept (concept is absent or negated) then label the note as a 0. If you are uncertain then label the note as a 2.<br><br>Here are some examples of 0, 1, and 2 labels:<br><br>#####<br>text: the patient was diagnosed with ptsd in 2019<br>label: 1<br><br>#####<br>text: the patient has post traumatic stress disorder<br>label: 1<br><br>#####<br>text: it is uncertain whether the patient has ptsd<br>label: 2 |

| | |
|---|---|
| | #####<br>text: patient has a history of post traumatic stress<br>label: 1<br><br>#####<br>text: there is no evidence of ptsd but the patient has a history of glioblastoma and crc<br>label: 0 |
| ADHD | For this task, we are labeling notes as ‘positive’ (with a 1) if they suggest the patient has had a history of ADHD. Here, ADHD is a broad term that can fall under a larger umbrella for a patient having a history of ADHD. We are not looking to fulfill any diagnostic clinical criteria, we want you to use your clinical intuition based on what you would label this note. We are choosing to go with a clinical diagnosis realizing that formal diagnosis criteria aren’t readily available in a patient’s notes. Here are some examples of terms that fall under our umbrella categorization of a patient having a history of ADHD: 'adolescent adhd', 'add', 'attentional deficit', etc…). If the note does not suggest the patient has a history of the concept (concept is absent or negated) then label the note as a 0. If you are uncertain then label the note as a 2.<br><br>Here are some examples of 0, 1, and 2 labels:<br><br>#####<br>text: the patient was diagnosed with adhd in 2019<br>label: 1<br><br>#####<br>text: the patient has add<br>label: 1<br><br>#####<br>text: it is uncertain whether the patient has attention deficit disorder<br>label: 2<br><br>#####<br>text: patient has a history of attention deficit hyperactivity disorder<br>label: 1<br><br>#####<br>text: there is no evidence of add but the patient has a history of glioblastoma and crc<br>label: 0 |
| Liver disease | For this task, we are labeling notes as ‘positive’ (with a 1) if they suggest the patient has had a history of liver disease. Here, liver disease is a broad term that can fall under a larger umbrella for a patient having a history of liver disease. We are not looking to fulfill any diagnostic clinical criteria, we want you to use your clinical intuition based on what you would label this note. We are choosing to go with a clinical diagnosis realizing that formal diagnosis criteria aren’t readily available in a patient’s notes. Here are some examples of terms that fall under our umbrella categorization of a patient having a history of liver disease: 'hepatitis e', 'hepatic encephalopathy', etc…). If the note does not suggest the patient has a history of the concept (concept is absent or negated) then label the note as a 0. If you are uncertain then label the note as a 2.<br><br>Here are some examples of 0, 1, and 2 labels: |

| | |
|---|---|
| | #####<br>text: the patient was diagnosed with nonalcoholic fatty liver disease in 2019<br>label: 1<br><br>#####<br>text: the patient has alcoholic hepatitis<br>label: 1<br><br>#####<br>text: it was uncertain whether the patient has hep c<br>label: 2<br><br>#####<br>text: patient has a history of chronic liver disease<br>label: 1<br><br>#####<br>text: there is no evidence of cirrhosis but the patient has a history of glioblastoma and crc<br>label: 0 |
| Suicidal behavior | For this task, we are labeling notes as ‘positive’ (with a 1) if they suggest the patient has had a history of suicidal behavior. Here, suicidal behavior is a broad term that can fall under a larger umbrella for a patient having a history of suicidal behavior. We are not looking to fulfill any diagnostic clinical criteria, we want you to use your clinical intuition based on what you would label this note. We are choosing to go with a clinical diagnosis realizing that formal diagnosis criteria aren’t readily available in a patient’s notes. Here are some examples of terms that fall under our umbrella categorization of a patient having a history of suicidal behavior: 'suicidal impulses', 'suicidal intent', 'active si', 'suicide ideation', etc…). If the note does not suggest the patient has a history of the concept (concept is absent or negated) then label the note as a 0. If you are uncertain then label the note as a 2.<br><br>Here are some examples of 0, 1, and 2 labels:<br><br>#####<br>text: the patient attempted suicide in 2019<br>label: 1<br><br>#####<br>text: the patient has active suicidal ideation<br>label: 1<br><br>#####<br>text: it was uncertain whether the patient was suicidal<br>label: 2<br><br>#####<br>text: patient has a history of si<br>label: 1<br><br>#####<br>text: there is no evidence of attempted suicide but the patient has a history of glioblastoma and crc<br>label: 0 |

| | |
|---|---|
| Chronic pain | For this task, we are labeling notes as ‘positive’ (with a 1) if they suggest the patient has had a history of chronic pain. Here, chronic pain is a broad term that can fall under a larger umbrella for a patient having a history of chronic pain. We are not looking to fulfill any diagnostic clinical criteria, we want you to use your clinical intuition based on what you would label this note. We are choosing to go with a clinical diagnosis realizing that formal diagnosis criteria aren’t readily available in a patient’s notes. Here are some examples of terms that fall under our umbrella categorization of a patient having a history of chronic pain: 'chronic back pain', 'chronic radiating pain', 'ongoing pain', 'long lasting pain', etc…). If the note does not suggest the patient has a history of the concept (concept is absent or negated) then label the note as a 0. If you are uncertain then label the note as a 2.<br><br>Here are some examples of 0, 1, and 2 labels:<br><br>#####<br>text: the patient has had chronic abd pain since 2019<br>label: 1<br><br>#####<br>text: the patient has persistent back pain<br>label: 1<br><br>#####<br>text: it was uncertain whether the patient has chronic neck pain<br>label: 2<br><br>#####<br>text: patient has a history of long lasting knee pain<br>label: 1<br><br>#####<br>text: there is no evidence of chronic pain but the patient has a history of glioblastoma and crc<br>label: 0 |
| Unemployment | For this task, we are labeling notes as ‘positive’ (with a 1) if they suggest the patient has had a history of unemployment. Here, unemployment is a broad term that can fall under a larger umbrella for a patient having a history of unemployment. We are not looking to fulfill any diagnostic clinical criteria, we want you to use your clinical intuition based on what you would label this note. We are choosing to go with a clinical diagnosis realizing that formal diagnosis criteria aren’t readily available in a patient’s notes. Here are some examples of terms that fall under our umbrella categorization of a patient having a history of unemployment: 'without work', 'jobless', 'not employed', 'seeking work', etc…). If the note does not suggest the patient has a history of the concept (concept is absent or negated) then label the note as a 0. If you are uncertain then label the note as a 2.<br><br>Here are some examples of 0, 1, and 2 labels:<br><br>#####<br>text: the patient has been unemployed since 2019<br>label: 1<br><br>#####<br>text: the patient is currently jobless |

| | |
|---|---|
| | label: 1<br><br>#####<br>text: it is uncertain whether the patient has a paying job<br>label: 2<br><br>#####<br>text: patient relies on unemployment benefits for money<br>label: 1<br><br>#####<br>text: the patient has a stable job but is still struggling financially<br>label: 0 |

| Supplementary Table 19: Stanford MOUD Zero-shot NER annotation instructions |
|---|
| Review the sentence and extract all clinically relevant entities that were mentioned in the text into a python list. Clinically relevant entities and concepts include a broad range of elements that are essential for understanding a patient's health status and making informed medical decisions. These entities can be categorized into several key types, including:<br>1. Diagnoses and diseases (diabetes, hypertension, or pneumonia)<br>2. Signs and symptoms (include observable indicators or patient-reported issues like fever, shortness of breath, or pain)<br>3. Medications (represent any prescribed drugs or treatments, such as insulin or antibiotics)<br>4. Procedures and interventions (include medical or surgical actions, such as biopsies or imaging scans<br>5. Laboratory and imaging (this includes results, significant lab values or findings from diagnostic tests, like elevated white blood cell counts or abnormal imaging results.)<br>6. Social determinants of health (SDoH) (such as housing instability or employment status)<br>7. Allergies and adverse reactions (including known allergies to medications like penicillin)<br>8. Lifestyle factors (such as smoking, alcohol use, diet, and exercise<br>9. Family and medical history (elements that provide context from the patient's personal and family health background, such as a history of cancer)<br>10. Patient demographics (including age, gender, and ethnicity)<br>Example task 1:<br>1. Sentence: “the patient 62yo male with is history of hypertension and chf they are currently homeless”<br>2. Label: [62yo, male, hypertension, chf, homeless]<br>Example task 2:<br>1. Sentence: “smoking status: never smoker”<br>2. Label: [never smoker]<br>Example task 3:<br>1. Sentence: “patient pmh for depression, ptsd, schizophrenia (vs bipolar vs illicit substance intoxication/withdrawal), polysubstance use (opiates, cocaine, meth, thc, baclofen/gabapentin overuse and prior overdose)”<br>2. Label: [depression, ptsd, schizophrenia, bipolar, illicit substance intoxication/withdrawal, polysubstance use, opiates, cocaine, meth, thc, baclofen/gabapentin, overdose] |

Supplementary Figure 1: Flow diagram depicting the datasets used throughout our experiments.

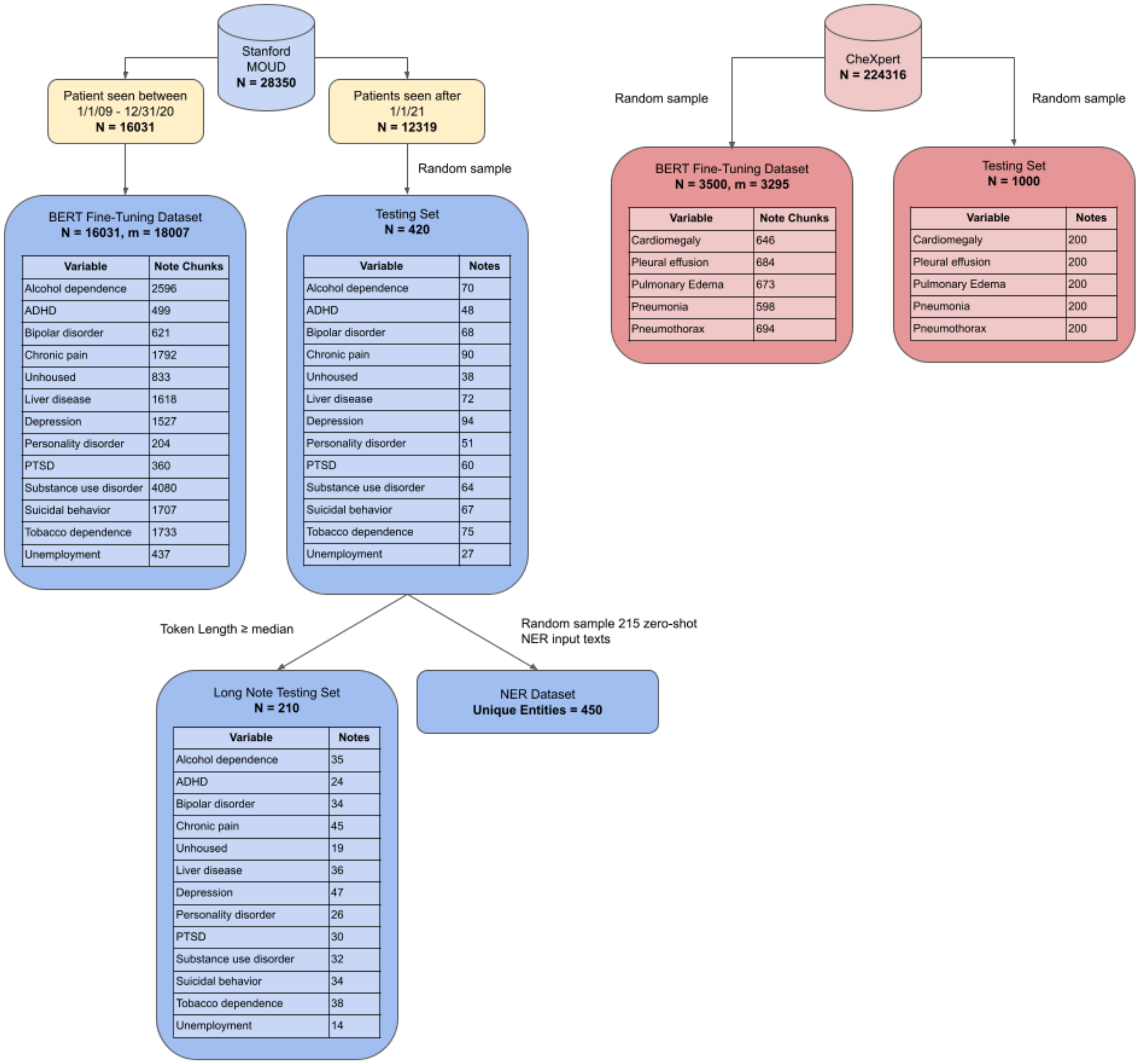


In this diagram, N represents unique notes, m represents unique note chunks.

Supplementary Figure 2: Zero-shot NER prompt templates for entity recognition step of CLEAR.

Zero-shot NER Prompts

| |
|---|
| catchAll: What are all the named entities in this text: {text} |
| medSocial: What are all the social determinants of health named entities in this text: {text} |
| clinical: What are all the clinical named entities in this text: {text} |
| medical: What are all the medical named entities in this text: {text} |

All four prompts were used for zero-shot NER and outputs were combined.

Supplementary Figure 3: LLM entity refinement prompt for entity selection step of CLEAR.

LLM Entity Refinement Prompt:

```
Given the following list of words, identify and return only those
words that are directly related to the term 'unhoused' within a
clinical or medical context. Consider terms that are synonyms,
acronyms, or are directly related to the term ‘unhoused’.
Present the output as a Python list like this: [output 1, output 2,
output 3, ...]. Here is an example: [unhoused, unsheltered,
houseless, etc…]
```

The target entity in this example is ‘unhoused’.

Supplementary Figure 4: LLM augmentation example prompt for entity selection step of CLEAR.

LLM Augmentation Prompt:

```
Please generate the most common synonyms a medical doctor
would use to describe ‘unemployment’ in medical literature or
clinician notes. Do not give me complete sentences, only words
and terms. Present the output as a Python list like this: [output
1, output 2, output 3, ...]. Here is an example: [unemployment,
not employed, seeking work, etc...]
```

The target entity in this example is ‘alcohol dependence’.

Supplementary Figure 5: LLM information extraction prompt.

Assertion Prompt

```
<|system|>: You are a helpful medical assistant. Label the text
with a 1 if the patient has a history of being unhoused, a 0 if
the patient does not have a history of being unhoused, or a 2 if
it is uncertain whether the patient has a history of being
unhoused. Here are some examples for the feature 'unhoused'
in the text:

#####
text: the patient has been homeless since 2019
label: 1
#####
text: the patient is currently unhoused
label: 1
#####
text: it is uncertain whether the patient is houseless
label: 2
#####
text: patient relies on transitional housing for shelter
label: 1
#####
text: the patient has stable housing but is struggling financially
label: 0
#####
<|prompter|>: text: {text}
<|assistant|>: label:
```

The target in this example is ‘unhoused’.

| Supplementary Table 20. Synonyms used to represent a concept for regular expression weak labeling functions. | |
|---|---|
| Concept | Synonyms |
| Cardiomegaly | 'pulmonary arterial hypertension', 'pulmonary congestion', 'enlarged heart', 'congestive heart failure', 'cardiac hypertrophy', 'left atrial enlargement', 'right sided cardiac failure', 'diastolic heart failure', 'pulmonary edema', 'acute cardiopulmonary disease', 'cardiac silhouette enlargement', 'enlarged', 'cardiomegaly', 'heart size', 'dilated cardiomyopathy', 'enlargement', 'left ventricular hypertrophy', 'diastolic chf', 'heart failure', 'cardiomyopathy', 'cardiac enlargement', 'acute cardiac decompensation', 'elevated pulmonary venous pressure', 'vascular congestion', 'enlargement of the cardiac silhouette', 'severe pulmonary hypertension', 'ventricular hypertrophy', 'chronic cardiomegaly', 'hypertrophy of the heart', 'right ventricular hypertrophy', 'heart enlargement', 'venous engorgement', 'enlarged cardiac silhouette', 'increased cardiac silhouette', 'pulmonary hypertension', 'big heart', 'central pulmonary vascular congestion', 'cardiac decompensation', 'enlarged pulmonary arteries' |
| Pleural effusion | 'loculated pleural effusion', 'pleural space', 'urinothorax', 'thoracic effusion', 'hemothorax', 'pleurisy with effusion', 'hydrothorax', 'bilateral pleural effusions', 'pleurodesis', 'low lung volumes', 'right pleural effusion', 'small right pleural effusion', 'pleural fluid excess', 'effusion', 'loculated right pleural effusion', 'lung atelectasis', 'pleural drain', 'water on the lung', 'layering pleural effusion', 'small right pleural effusions', 'aspiration', 'bilateral pleural effusion', 'chylothorax', 'left pleural effusion', 'small bilateral effusions', 'pneumothorax', 'small left pleural effusion', 'pleural fluid', 'pleural effusions', 'hydropneumothorax', 'bilateral effusions', 'chest fluid', 'multifocal pneumonia', 'small effusions', 'pleural effusion', 'small effusion', 'moderate pleural effusion', 'layering effusions', 'pyothorax', 'accumulation of fluid in pleura', 'lower lobe pneumonia', 'serous effusion', 'fluid in the pleura', 'layering pleural effusions', 'small pleural effusion', 'pleuritic effusion', 'interstitial edema', 'left effusion', 'effusions', 'small pleural effusions', 'large left pleural effusion', 'left pleural effusions', 'lower lobe atelectasis', 'pleural fluid collection' |
| Pulmonary Edema | 'edema', 'lung water', 'non-cardiogenic pulmonary edema', 'pneumonia', 'acute respiratory distress syndrome', 'bibasilar opacities', 'interstitial pulmonary edema', 'superimposed pulmonary edema', 'bilateral pleural effusion', 'heart failure', 'noncardiogenic edema', 'upper lobe pulmonary vascular congestion', 'pleural fluid', 'neurogenic pulmonary edema', 'bilateral effusions', 'interstitial lung edema', 'acute pulmonary edema', 'interstitial edema', 'wet lung', 'pulmonary hypertension', |

| | |
|---|---|
| | 'hape', 'moderate bilateral pleural effusions', 'overt pulmonary edema', 'pulmonary hemorrhage', 'effusion', 'pulmonary edema', 'permeability edema', 'mild pulmonary edema', 'pul edema', 'parenchymal opacities', 'fluid in lungs', 'lung opacification', 'high altitude pulmonary edema', 'pulm edema', 'vascular congestion', 'ards', 'pleural effusion', 'interstitial lung disease', 'pulmonary and interstitial edema', 'signs for overt pulmonary edema', 'central pulmonary vascular congestion', 'minimal pulmonary interstitial edema', 'ali', 'chf exacerbation', 'pulmonary consolidation', 'hilar congestion', 'hydrothorax', 'hypoxic respiratory failure', 'lung congestion', 'diastolic heart failure', 'flash pulmonary edema', 'basal interstitial edema', 'cardiomegaly', 'signs pulmonary edema', 'interstitial opacity', 'pulmonary vascular engorgement', 'diastolic chf', 'acute lung injury', 'chronic heart failure', 'mild fluid overload', 'hydrostatic pulmonary edema', 'elevated pulmonary venous pressure', 'worsening pulmonary edema or infiltrate', 'cardiogenic edema', 'with acute pulmonary edema', 'perihilar and interstitial edema', 'dilated pulmonary arteries', 're expansion pulmonary edema', 'pneumonitis', 'toxic pulmonary edema', 'respiratory distress', 'cardiac decompensation', 're expansion edema', 'respiratory failure', 'alveolar edema', 'pulmonary congestion', 'bibasilar atelectasis', 'mild to moderate pulmonary edema', 'mild pulmonary interstitial edema', 'pulmonary interstitial edema', 'post obstructive pulmonary edema', 'asymmetric pulmonary edema', 'plulmonary edema', 'mild edema', 'cardiogenic lung edema', 'pulmonary vascular congestion', 'interstitial opacities', 'pleural effusions', 'worsening pulmonary edema', 'parenchymal opacity', 'peripheral edema', 'persistent pulmonary vascular congestion', 'lung opacities', 'fluid overload', 'moderate pleural effusion', 'decompensated chf' |
| Pneumonia | 'radiographic pneumonia', 'walking pneumonia', 'aspiration pneumonia', 'infectious pneumonitis', 'cryptogenic organizing pneumonia', 'pleuropneumonia', 'pneumonia', 'bacterial lower respiratory infection', 'community-acquired pneumonia', 'bronchopneumonia', 'pulmonary infiltrates', 'hospital-acquired pneumonia', 'viral pneumonia', 'bacterial pneumonia', 'nosocomial pneumonia', 'atypical pneumonia', 'ventilator-associated pneumonia', 'lobar pneumonia', 'viral lower respiratory infection', 'interstitial pneumonia', 'fungal pneumonia', 'necrotizing pneumonia', 'pneumonitis', 'acute respiratory infection', 'primary atypical pneumonia', 'pulmonary infection', 'pna' |
| Pneumothorax | 'bilateral pneumothoraces', 'basilar pneumothorax', 'hydropneuthorax', 'with pneumothorax', 'air in pleural space', 'chest air pocket', 'thoracentesis', 'left lower lobe collapse', |

| | |
|---|---|
| | 'hydrothorax', 'left sided pneumothorax', 'right apical pneumothorax', 'complete lung collapse', 'persistent small apical left pneumothorax', 'postoperative pneumothorax', 'right pleural effusion', 'effusion', 'small right pleural effusion', 'loculated right pleural effusion', 'evaluate for pneumothorax', 'lung collapse', 'pulmonary collapse', 'hydro pneumothorax', 'pneumothoraces', 'thoracostomy tube', 'persistent bilateral pneumothorax', 'airleak', 'primary pneumothorax', 'pleural catheter', 'thoracic air', 'left pleural effusion', 'secondary pneumothorax', 'spontaneous pneumothorax', 'left apical pneumothorax', 'apical ptx', 'minimal apical right pneumothorax', 'pleurx catheter', 'pneumothorax', 'small left pleural effusion', 'hydropneumothorax', 'pleural effusions', 'air leak', 'air', 'intrathoracic air', 'complete collapse', 'compressive atelectasis', 'apical pneumothorax line', 'pleural effusion', 'air outside lung', 'displaced rib fractures', 'bilateral apical pneumothoraces', 'right pleural tube', 'left hydro pneumothorax', 'trapped lung', 'atelectasis', 'left pneumothorax', 'tension', 'pleural tubes', 'collapsed lung', 'lung deflation', 'ptx', 'effusions', 'subpulmonic pneumothorax', 'pleural cavity air', 'pleural air', 'traumatic pneumothorax', 'hemothorax', 'apical pneumothorax', 'pnx', 'persistent air leak', 'right pneumothorax', 'tension pneumothorax', 'subcutaneous emphysema', 'ptx' |

| Supplementary Table 21: Bio+Clinical BERT Fine-tuning Dataset | | | | | |
|---|---|---|---|---|---|
| Variable | Present | Negated/Absent | Uncertain | Total Chunks | LLM Labeler |
| Stanford MOUD Dataset | | | | | |
| Alcohol dependence | 820 | 1776 | 18 | 2614 | Flan-UL2 |
| ADHD | 76 | 423 | 1 | 500 | Mixtral |
| Bipolar disorder | 377 | 244 | 167 | 788 | Mixtral |
| Chronic pain | 1596 | 196 | 1 | 1793 | Flan-T5 |
| Unhoused | 296 | 537 | 1 | 834 | Flan-T5 |
| Liver disease | 1059 | 559 | 7 | 1625 | Flan-UL2 |
| Depression | 1322 | 205 | 191 | 1718 | Mixtral |
| Personality disorder | 60 | 144 | 0 | 204 | Llama-3 |
| PTSD | 175 | 185 | 7 | 367 | Llama-3 |
| Substance use disorder | 2873 | 1207 | 674 | 4754 | Mixtral |
| Suicidal behavior | 604 | 1103 | 16 | 1723 | Flan-T5 |
| Tobacco dependence | 850 | 883 | 12 | 1745 | Flan-UL2 |
| Unemployment | 300 | 137 | 1 | 438 | Flan-T5 |
| CheXpert Dataset | | | | | |
| Cardiomegaly | 432 | 214 | 54 | 646 | Flan-T5 |
| Pleural effusion | 487 | 197 | 16 | 684 | Flan-T5 |
| Pulmonary Edema | 363 | 310 | 27 | 673 | Flan-T5 |
| Pneumonia | 205 | 393 | 102 | 598 | Flan-T5 |
| Pneumothorax | 134 | 560 | 6 | 694 | Flan-T5 |
| For each variable, we selected the best-performing LLM, excluding GPT-4, to weakly label the fine-tuning dataset, as OpenAI's terms prohibit using GPT-4 outputs to develop competitor models. | | | | | |

Supplementary Figure 6: Chunk embedding RAG approach to information retrieval.

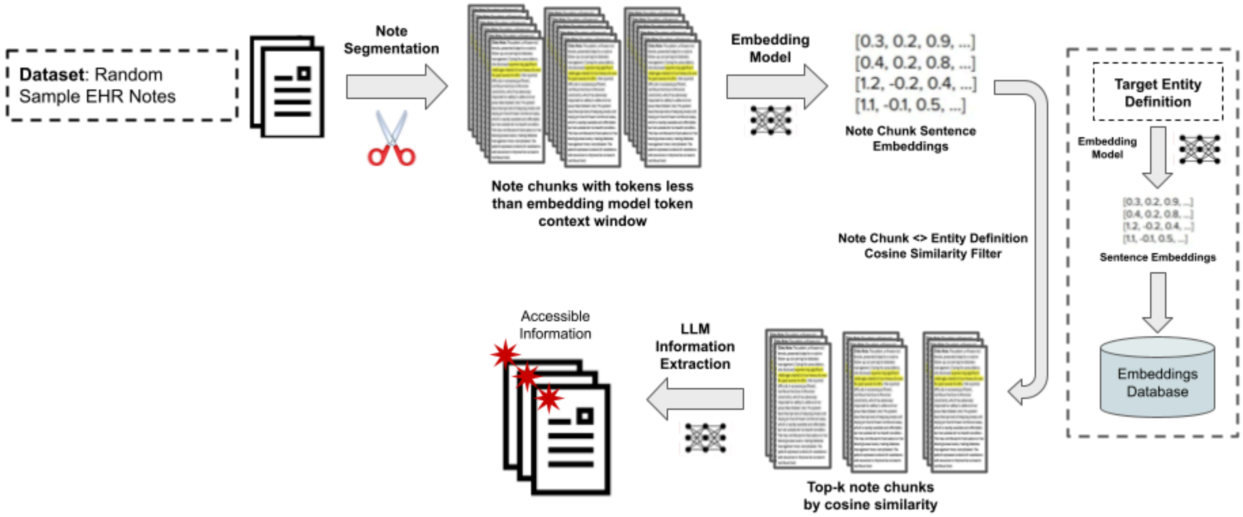


Chunk embedding RAG approach is upstream to LLM information extraction.

Supplementary Figure 7: Full note approach to information retrieval.

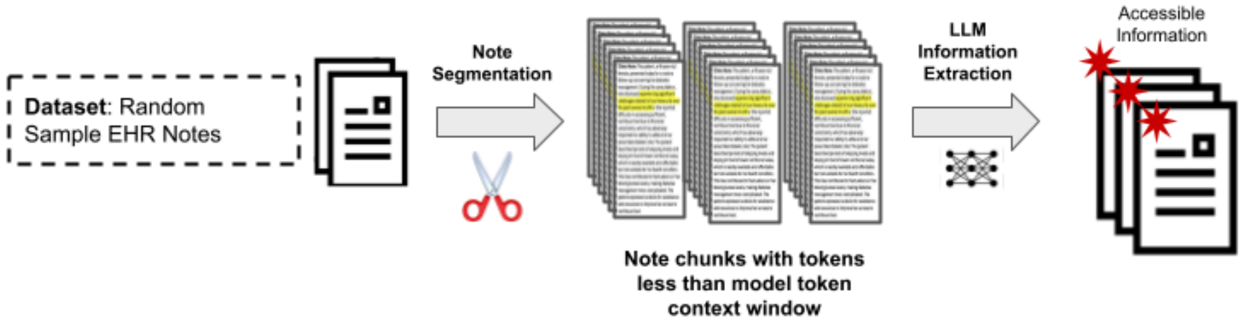


Full note approach is upstream to LLM information extraction.

| Supplementary Table 22: Overview of all models used across different tasks. | |
|---|---|
| Task | Models |
| NER | Flan-T5-XXL |
| LLM Augmentation | GPT-4 |
| Entity Selection:<br>Cosine Similarity | Bio+ClinicalBERT |
| Entity Selection:<br>LLM Filtering | GPT-4 |
| Information Extraction | Med42-70b, Mixtral-8x7B-Instruct-v0.1, Llama-3-70b, Flan-T5-XXL, Flan-UL2, and GPT-4 |
| Chunk Embedding Model | BAAI Generalized Embeddings Large English v1.5 |
| Model Distillation | Bio+ClinicalBERT |

| Supplementary Table 23: Parameters for NER and information extraction models | | |
|---|---|---|
| Parameter | NER Model | Information Extraction Models |
| Seed | 1234 | 1234 |
| Max time | 300 | NA |
| No repeat ngram size | 3 | NA |
| Repetition penalty | 2.5 | NA |
| Top-p | 0.9 | 0.2 |
| Temperature | 0.2 | 0.2 |
| Max new tokens | 1024 | 4 |
| Do sample | NA | False |
